# Cytosine-5 RNA methylation links protein synthesis to cell metabolism

**DOI:** 10.1371/journal.pbio.3000297

**Published:** 2019-06-14

**Authors:** Nikoletta A. Gkatza, Cecilia Castro, Robert F. Harvey, Matthias Heiß, Martyna C. Popis, Sandra Blanco, Susanne Bornelöv, Abdulrahim A. Sajini, Joseph G. Gleeson, Julian L. Griffin, James A. West, Stefanie Kellner, Anne E. Willis, Sabine Dietmann, Michaela Frye

**Affiliations:** 1 Department of Genetics, University of Cambridge, Cambridge, United Kingdom; 2 Department of Biochemistry, University of Cambridge, Cambridge, United Kingdom; 3 Medical Research Council Toxicology Unit, University of Cambridge, Cambridge, United Kingdom; 4 Department of Chemistry, Ludwig-Maximilians-University Munich, Munich, Germany; 5 Cancer Cell Signaling and Metabolism Lab, Proteomics Unit CIC bioGUNE, Derio, Spain; 6 Molecular Mechanisms Program, Centro de Investigación del Cáncer and Instituto de Biología Molecular y Celular del Cáncer, Consejo Superior de Investigaciones Científicas (CSIC)-University of Salamanca, Salamanca, Spain; 7 Wellcome–Medical Research Council Cambridge Stem Cell Institute, University of Cambridge, Cambridge, United Kingdom; 8 Department of Biomedical Engineering, Khalifa University of Science and Technology, Abu Dhabi, United Arab Emirates; 9 Department of Neurosciences, San Diego School of Medicine, University of California, La Jolla, California, United States of America; 10 German Cancer Center (Deutsches Krebsforschungszntrum), Heidelberg, Germany; Case Western Reserve University, UNITED STATES

## Abstract

Posttranscriptional modifications in transfer RNA (tRNA) are often critical for normal development because they adapt protein synthesis rates to a dynamically changing microenvironment. However, the precise cellular mechanisms linking the extrinsic stimulus to the intrinsic RNA modification pathways remain largely unclear. Here, we identified the cytosine-5 RNA methyltransferase NSUN2 as a sensor for external stress stimuli. Exposure to oxidative stress efficiently repressed NSUN2, causing a reduction of methylation at specific tRNA sites. Using metabolic profiling, we showed that loss of tRNA methylation captured cells in a distinct catabolic state. Mechanistically, loss of NSUN2 altered the biogenesis of tRNA-derived noncoding fragments (tRFs) in response to stress, leading to impaired regulation of protein synthesis. The intracellular accumulation of a specific subset of tRFs correlated with the dynamic repression of global protein synthesis. Finally, NSUN2-driven RNA methylation was functionally required to adapt cell cycle progression to the early stress response. In summary, we revealed that changes in tRNA methylation profiles were sufficient to specify cellular metabolic states and efficiently adapt protein synthesis rates to cell stress.

## Abstract 2

The dynamic and site-specific 5-methylation of cytosine in tRNAs can act as a sensor of oxidative stress, ensuring the fast adaptation of protein synthesis rates to temporary cellular metabolic requirements.

## Introduction

Clinical and genetic heterogeneity in diseases such as metabolic disorders and cancer remains a major challenge for targeted therapies. Phenotypic disease variation can be caused by cell type–specific modulation of gene products via both the transcription and translation machineries [1,2]. Recently, the formation of a variety of chemical modifications in RNA emerged as an additional regulatory layer of gene expression programmes [3].

Of the over 170 known RNA modifications, methylation is the most common [4]. In RNA, 5-methylcytosine (m^5^C) is often required for normal development, and its formation is mediated by at least eight highly conserved enzymes (NSUN1–7 and DNMT2) [5]. For instance, loss-of-function mutations in the human *NSUN2* and *NSUN3* genes cause neurodevelopmental and mitochondrial disorders, respectively [6–9]. NSUN2 methylates transfer RNAs (tRNAs) site-specifically at either the anticodon or variable loop (VL), thereby protecting tRNAs from endonucleolytic cleavage [10,11]. This protection is important to prevent the accumulation of tRNA-derived fragments (tRFs), which would otherwise inhibit protein synthesis [11–13].

Phenotypically, the loss of NSUN2 thus leads to an expansion of low translating stem and progenitor populations in skin and brain [11,12,14]. Low protein synthesis in slowly cycling or quiescent stem cells saves energy and prevents premature exhaustion [12,15–17] and is required to maintain a fully functional stem cell state by enhancing resilience towards differentiation cues [12].

The precise mechanisms linking external stresses to RNA modifications and protein synthesis have remained largely unknown in mammals. Here, we reveal that one such mechanism is the endogenous and dynamic methylation of RNA by NSUN2. Our data show that expression of NSUN2 is required to metabolically support high protein synthesis rates. By performing a time course that mapped endogenous, stress-induced tRNA methylation, we observed for the first time, to our knowledge, that loss of methylation occurs in a highly site-specific manner as early as 2 hours following an insult. Stress-induced loss of tRNA methylation altered both the fragmentation pattern among tRNAs and protein synthesis rates. Thus, our data reveal how RNA methylation is mechanistically integrated into metabolic homeostasis.

## Results

### NSUN2 regulation of stem cell differentiation is not controlled via transcription

**Fig 1:**
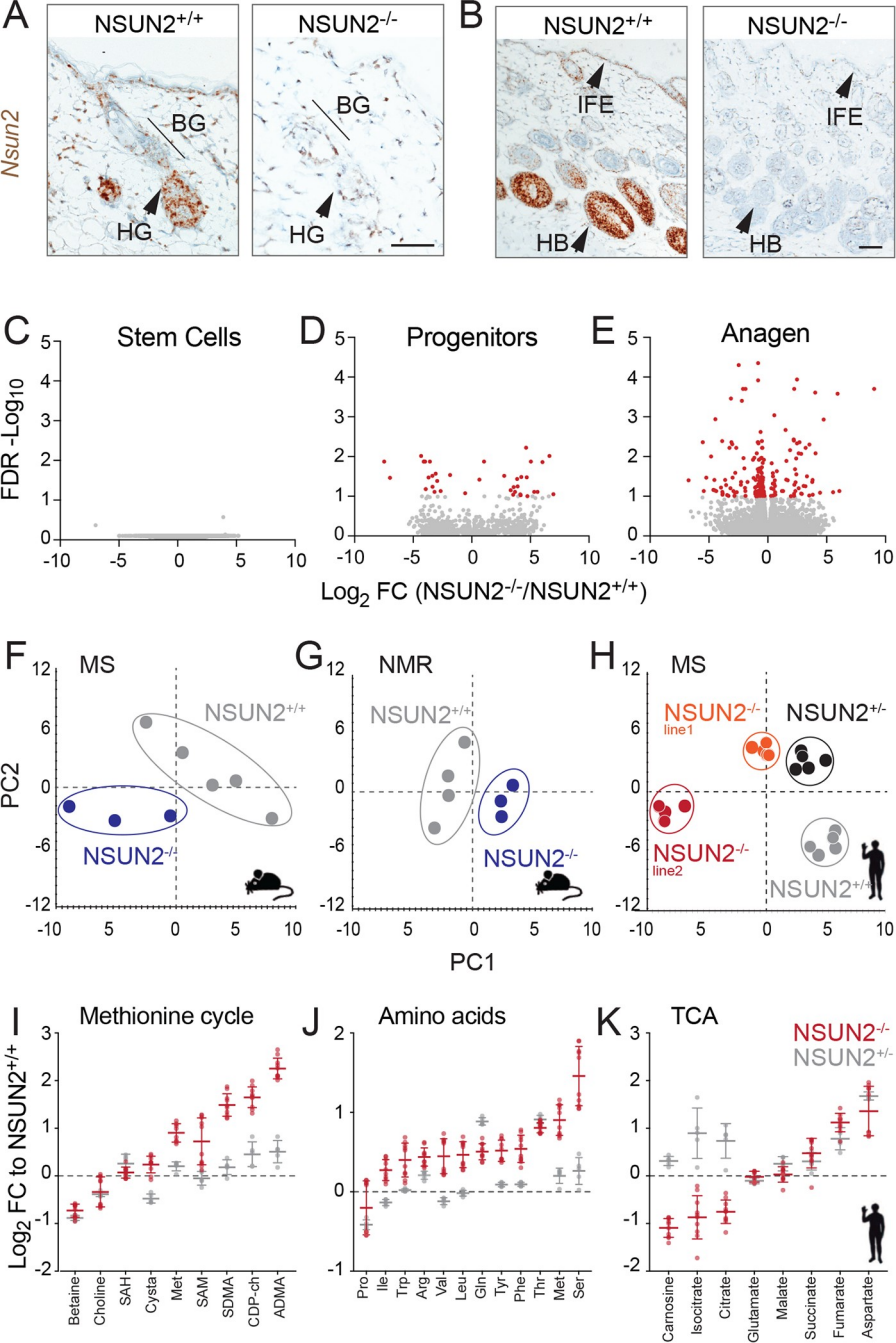
Loss of NSUN2 triggers a shift of the metabolic state towards catabolism. (A, B) Detection of *Nsun2* RNA in *Nsun2*+/+ and *Nsun2−*/*−* mouse skin in early (A) and late (B) anagen. Scale bar: 50 μm. (C-E) Transcriptional changes in skin of wild-type (*Nsun2*+/+) and *Nsun2* knockout (*Nsun2−*/*−*) mice. Highlighted in red are significant FC expression differences (FDR < 0.05) in hair follicle stem cells (CD34+/ITGA6^high^) (C), progenitor cells (PCAD^high^/ITGA6^low^) (D), and anagen skin (E). (*n* = 3–4 mice per genotype and condition). (F-H) Multivariate analyses of data obtained from MS (F) or NMR spectroscopy–based metabolic profiling (G) using mouse back skin (*n* = 3–5 mice) or human dermal fibroblasts (*n* = 5 samples per genotype) (H). Model parameters: R^2^X = 94.5%, R^2^Y = 99.9%, and Q^2^ = 95.8% using partial least square discriminant analysis (F), R^2^X = 70% and Q^2^ = 30% (G), and R^2^X = 85.9% and Q^2^ = 78.6% (H), using principal component analysis. (I-K) Metabolic differences between *NSUN2*+/− and *NSUN2*−/− normalised to *NSUN2*+/+ human dermal fibroblasts relating to the methionine cycle (I), free amino acids (J), and the TCA cycle (K). The underlying data for this figure can be found in S1–S3 Data and S1 File. BG, hair follicle bulge; FC, fold-change; FDR, false discovery rate; HB, hair bulb; IFE, interfollicular epidermis; ITGA6, integrin alpha-6; MS, mass spectrometry; NMR, nuclear magnetic resonance; PC1, Principal Component 1; PC2, Principal Component 2; PCAD, P-cadherin; TCA, tricarboxylic acid.

Disruption of the *Nsun2* gene in mice causes global hypomethylation of tRNAs and a developmental growth retardation [5]. The abnormal development of tissues including brain and skin is the result of impaired stem cell differentiation [11,14,18] (S1A and S1B Fig). The expression of NSUN2 is highly dynamic within tissues. For instance, NSUN2 is absent in quiescent stem cells in hair follicle bulges (BGs), steadily increases in progenitor cells in the hair germ (HG), and is highest in the growing (anagen) hair bulb (HB) (Fig 1A and 1B).

To dissect the underlying molecular pathways that decrease stem cell sensitivity towards differentiation stimuli in the absence of NSUN2, we transcriptionally profiled mouse stem and progenitor cell populations in the hair follicles, as well as hair follicles in the resting (telogen) or growing (anagen) state. Stem and progenitor cells were isolated by flow sorting using the cell surface marker integrin alpha-6 (ITGA6) and then further separated into quiescent stem cells (ITGA6^high^/CD34^+^) and activated, cycling progenitors (ITGA6^low^/P-cadherin [PCAD]^+^) (S1B–S1D Fig) [19–21]. In *Nsun2*−/− mouse skin, quiescent stem cells increase at the expense of activated progenitors (S1B Fig) [12,18]. Transcriptional comparison of stem and progenitor cells isolated from wild-type and *Nsun2*−/− mice revealed that this increase does not appear to be transcriptionally driven (Fig 1C and 1D). Transcriptionally, these cell populations were highly similar, regardless of their expression of NSUN2 (S1 Data).

Similarly, loss of NSUN2 in actively growing hair follicles (anagen) that highly express NSUN2 in wild-type cells (Fig 1B) resulted in the differential expression of just over 100 genes (red) (Fig 1E). Metabolic genes were highly enriched in these few differentially expressed genes, suggesting that changes in metabolism might account for the insensitivity of stem cells to differentiation cues (S1E and S1F Fig).

### NSUN2 regulates metabolism by promoting an anabolic cell state

NSUN2 methylates most tRNAs and a smaller number of noncoding and coding RNAs using S-adenosyl-methionine (SAM) as the methyl donor (S1G Fig) [5,22–24]. SAM and its downstream metabolite, S-adenosyl-homocysteine (SAH), are integral to the one-carbon metabolism encompassing the folate and methionine cycles (S1H Fig). The one-carbon metabolism supports multiple physiological processes, including nucleotide biosynthesis (purines and thymidine), amino acid homeostasis, and the redox defence system [25]. Similar to NSUN2 deletion, dysregulation of the one-carbon metabolism impairs foetal growth and has been linked to neurodevelopmental disorders and cancer [25–27].

We therefore asked whether NSUN2 controlled the one-carbon metabolic cycles that generate the methyl donor required for NSUN2 to modify RNA. We used mass spectrometry (MS) and nuclear magnetic resonance (NMR) spectroscopy to measure how metabolites were affected by loss of NSUN2. We compared wild-type and *Nsun2−*/*−* mouse skin in anagen, as well as human dermal fibroblasts expressing (*NSUN2*+/+ and *NSUN2*+/*−*) or lacking NSUN2 (*NSUN2−*/*−*) (S2 Data) [7]. Multivariate analysis of either the MS or NMR spectroscopic data clearly separated the genotypes (Fig 1F–1H). Therefore, loss of NSUN2 established a distinct cellular metabolic state.

Three major metabolic pathways were affected by deletion of NSUN2: (1) the methionine cycle, (2) amino acid synthetic pathways, and (3) the tricarboxylic acid (TCA) cycle (Fig 1I–1K; S1I–S1N Fig; S2 Data; S3 Data). First, higher levels of metabolites of the methionine cycle strongly indicated that protein degradation was enhanced in the absence of NSUN2. Cellular metabolism up-regulated methionine and SAM, but not SAH, in response to loss of NSUN2 (Fig 1I). Such an alteration in the stochastic ratio of SAM to SAH can reshape the landscape of protein methylation [28]. In particular, increased levels of free symmetric dimethylarginine (SDMA) and asymmetric dimethylarginine (ADMA) can only be caused by enhanced protein degradation, because they are generated solely upon proteolysis of methylated proteins [29]. Second, we found substantial enhancement of free amino acid levels in the absence of NSUN2 (Fig 1J; S1J and S1M Fig), indicating a reduced rate of protein synthesis. Third, the changes we observed in the metabolites of the TCA cycle indicated that upon loss of NSUN2, metabolism rebalanced from oxidative phosphorylation towards glycolysis (Fig 1K).

In conclusion, the metabolic changes we observed included increased protein degradation, suppressed protein synthesis, and enhanced glycolysis and together showed that cells lacking NSUN2 are maintained in a catabolic state (S1O Fig).

### Methylation-dependent and -independent NSUN2 functions regulate metabolism

**Fig 2:**
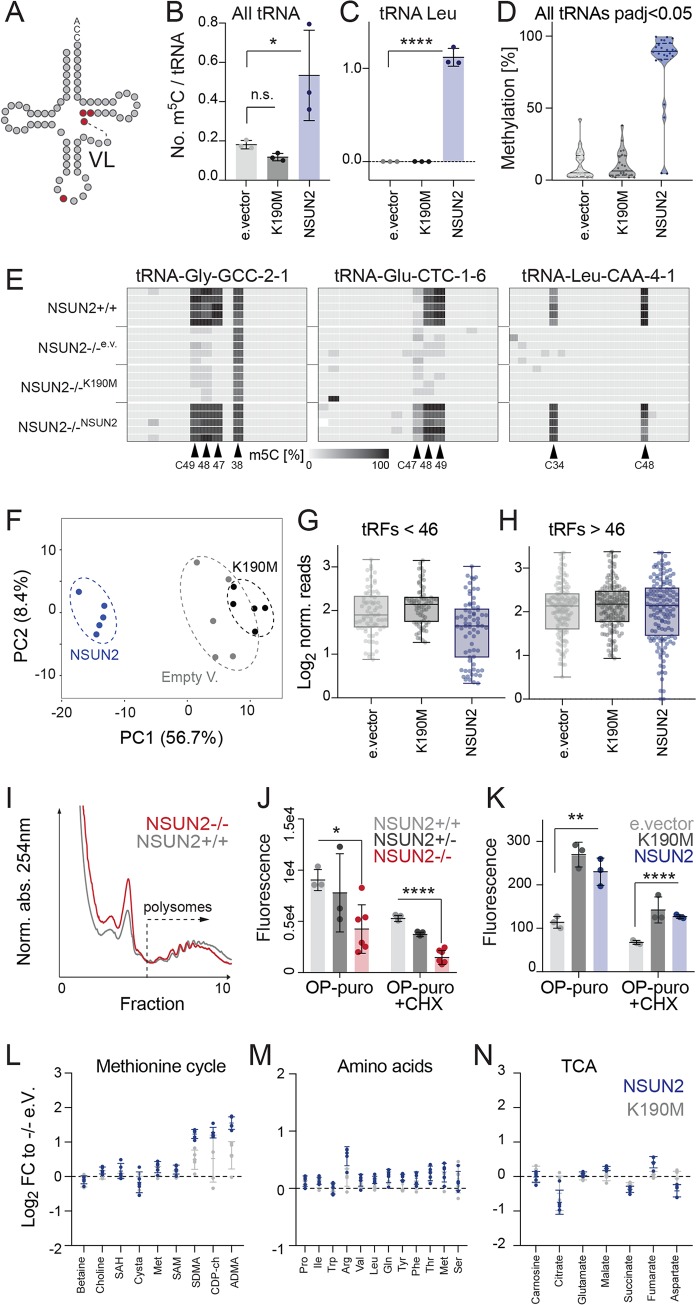
Methylation-dependent and -independent functions of NSUN2. (A) Schematic representation of NSUN2-methylated tRNA sites in the anticodon loop (C34) and the VL (C46, C47). (B, C) Number of m^5^C per tRNA in all tRNAs (B) or tRNA leucine (C) quantified by mass spectrometry in *NSUN2−*/ cells reexpressing NSUN2, the enzymatic dead version of NSUN2 (K190M), or the empty (‘e.’) vector control. *padj < 0.05; ****padj < 0.0001 (ordinary one-way ANOVA, multiple comparisons). (D) Quantification of m^5^C levels in all rescued tRNAs (*p*adj < 0.05) using RNA bisulfite sequencing. (E) Heatmaps of example tRNAs showing the rescued m^5^C sites in five replicates of *NSUN2*+/+ cells or *NSUN2−/−* cells reexpressing NSUN2, K190M, or the empty vector (‘e.v.’). (F) PCAs of tRFs differentially abundant in NSUN2-overexpressing *NSUN2−*/*−* cells. (G, H) Log_2_ coverage of tRFs smaller than 46 nucleotides (G) or larger than 46 nucleotides (H). (I) Polysome profile of NSUN2-expressing (*NSUN2*+/+) and -lacking (*NSUN2−*/*−*) cells. Shown is one out three replicates. (J, K) Protein synthesis levels measured by flow cytometry using OP-puro in the indicated cells. CHX served as a control. Data represent mean, and error bars are ±SD. Student’s *t* test. *p < 0.05, **p < 0.01, ****p < 0.0001. (L-N) Metabolic differences between *NSUN2−*/*−* cells overexpressing the NSUN2 or K190 protein normalised (‘norm.’) to *NSUN2−*/*−* cell infected with the empty vector control (‘e.V.’) relating to the methionine cycle (L), free amino acids (M), and the TCA cycle (N). The underlying data for this figure can be found in S5–S7 Data and S1 File. ADMA, asymmetric dimethylarginine; CHX, cycloheximide; FC, fold-change; m^5^C, 5-methylcytosine; OP-puro, O-propargyl-puromycin; PCA, principle component analysis; SAH, S-adenosyl-homocysteine; SAM, S-adenosyl-methionine; SDMA, symmetric dimethylarginine; TCA, tricarboxylic acid; tRF, tRNA-derived fragment; tRNA, transfer RNA; VL, variable loop.

NSUN2-expressing and -lacking mouse epidermal cell populations were metabolically different, but their transcriptomes were highly similar. In contrast, RNA sequencing (RNA-seq) of human NSUN2-expressing and -lacking dermal fibroblasts identified 2,867 differentially expressed genes (S2A and S2B Fig; S4A Data). However, the transcriptome of *NSUN2−*/*−* cells remained largely unaltered when we reexpressed the NSUN2 protein (S2C–S2E Fig; S4B Data), indicating that the differences in gene expression were cell line–specific rather than driven by the presence or absence of NSUN2. To confirm that we indeed rescued tRNA methylation, we reexpressed the wild type (NSUN2), an enzymatic dead version of NSUN2 (K190M), or the empty vector (e.vector) as a control in *NSUN2−*/*−* cells [30]. Quantitative MS and RNA bisulfite sequencing (BS-seq) confirmed remethylation of NSUN2-specific sites in tRNAs (Fig 2A–2E; S5 Data).

One functional role of m^5^C in tRNAs is to protect from endonucleolytic cleavage by angiogenin [10,11]. To measure the production of tRFs, we performed small RNA-seq in the rescued *NSUN2*−/− cells. Next, we identified all significantly different tRFs between K190M- and NSUN2-expressing cells (*p*adj < 0.05) that were unaltered when K190M was compared to the empty vector control (*p*adj > 0.75) (Fig 2F; S6 Data). Overexpression of the wild-type but not the enzymatic dead NSUN2 protein rescued the formation of tRFs smaller than 46 nucleotides (Fig 2G and 2H), demonstrating that the biogenesis of distinct tRFs was driven by NSUN2-specific methylation.

Angiogenin-mediated tRNA cleavage inhibits global protein synthesis [31]. Therefore, we next asked how mRNA translation was affected by removal of NSUN2. Polysome profiling confirmed differences in the global abundance of polysomes following NSUN2-removal (Fig 2I; S2F Fig). To quantify de novo protein synthesis, we measured the incorporation of the reporter molecule O-propargyl-puromycin (OP-puro) into nascent polypeptides (S2G Fig) [32]. OP-puro forms covalent conjugates that can be imaged by microscopy and quantified by flow cytometry (S2H Fig). Treatment with cycloheximide (CHX), a potent inhibitor of mRNA translation, served as a positive control [33]. Deletion of NSUN2 repressed protein synthesis (Fig 2J), whereas inhibition of angiogenin was sufficient to up-regulate protein synthesis (S2I Fig). Inhibition of mammalian target of rapamycin complex 1 (mTORC1) using rapamycin down-regulated NSUN2 protein expression but reduced protein synthesis similarly in NSUN2-expressing and -lacking cells (S2J–S2L Fig). Thus, loss of NSUN2 led to decreased protein synthesis rate. Accordingly, reexpression of NSUN2 enhanced de novo protein synthesis, but this effect was independent of its methylation activity (Fig 2K). One explanation for why expression of K190M also led to the up-regulation of protein synthesis might be that its binding protected the unmethylated tRNAs from processing.

Finally, we asked whether restoring NSUN2-specific methylation sites also affected the one-carbon metabolism. Although reexpression of NSUN2 or K190M significantly altered the metabolic profile of *NSUN2−/−* cells (S2M and S2N Fig), NSUN2 expression was not sufficient to reverse the catabolic cell state (Fig 2L–2N; S7 Data).

Together, NSUN2-dependent methylation protected tRNAs from processing into tRFs, and reexpression of NSUN2 in *NSUN2−*/*−* cells enhanced protein synthesis in a methylation-independent manner. However, expression of NSUN2 was not sufficient to switch from a catabolic to anabolic cell state.

### NSUN2 functions in the dynamic adaptation of protein synthesis in response to stress

One explanation for why up-regulation of NSUN2 was not sufficient to induce an anabolic cell state was that both tRNA methylation and metabolism normally function in response to external cues. Therefore, we next sought to determine the importance of NSUN2-mediated RNA methylation in response to a changing microenvironment. As a stimulus, we chose stress because NSUN2 is required for the proper cellular response upon stress signals in brain and skin in vivo [11,12].

**Fig 3:**
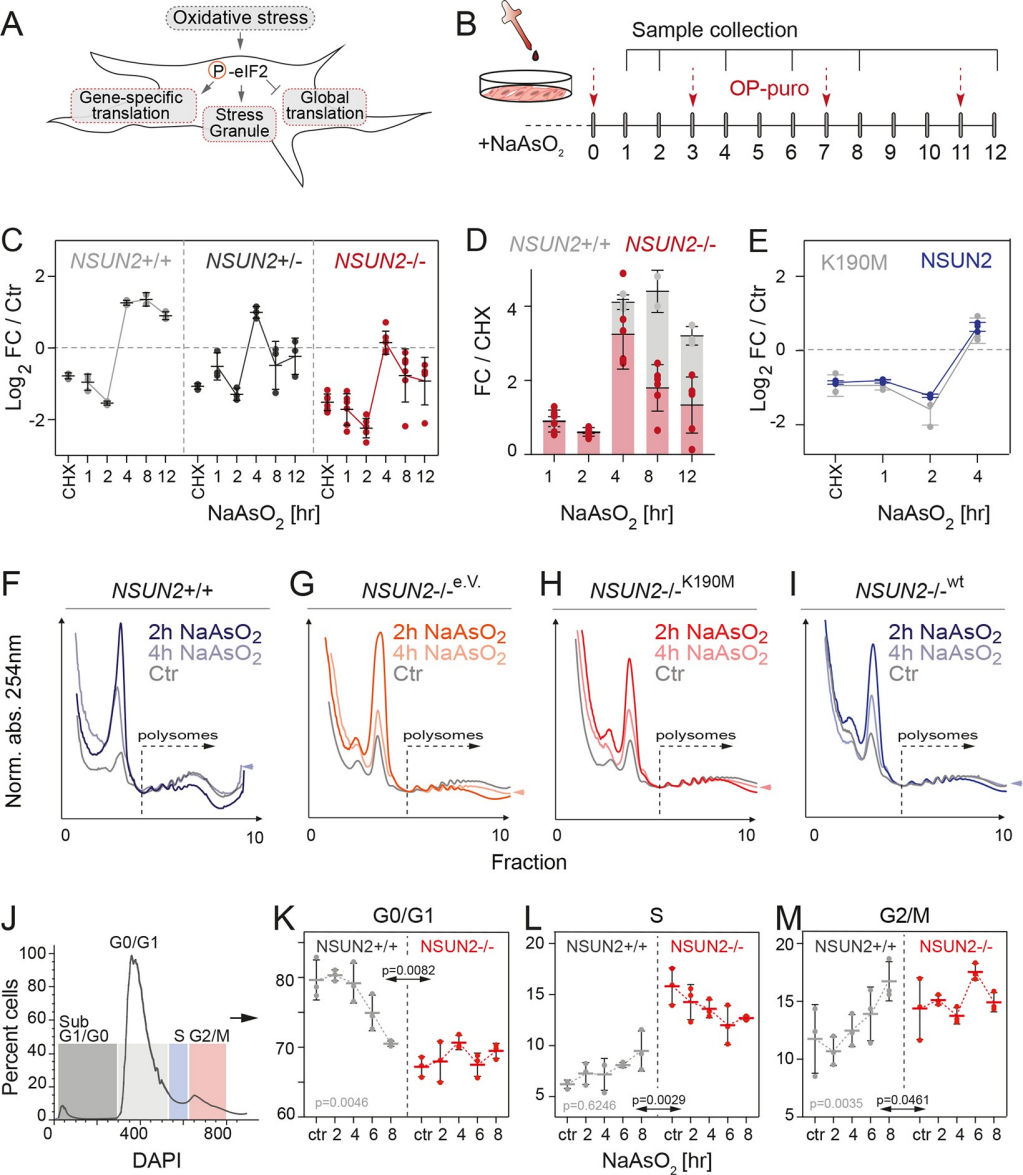
NSUN2 functions in the cell cycle to adapt dynamic protein synthesis in response to stress. (A) Schematic representation how oxidative stress modulates global and gene-specific translation. (B) Treatment regime using arsenite to induce stress and OP-puro to measure protein synthesis. (C) Log_2_ FC of protein synthesis in *NSUN2*+/+, *NSUN2*+/− and *NSUN2*−/− cells in response to stress compared to the untreated controls (‘Ctr’). CHX served as a control. (*n* = 2–3 samples per time point). (D) Relative protein synthesis levels in response to stress in *NSUN2*+/+ and −/− cells measured as FC compared to CHX control. (E) Log_2_ FC of protein synthesis in *NSUN2*−/− cells rescued with NSUN2 or the enzymatic dead version K190M after exposure to stress at the indicated time points. (F-I) Polysome profile of *NSUN2*+/+ (F) and *NSUN2*−/− cells rescued with wt (I) or mutated NSUN2 (K190M) (H). The empty vector (‘e.V.’)-infected cells served as control (G). Shown is one out of two replicates. (J) Gating used for cell cycle analyses using DAPI incorporation. (K-M) Percentage of *NSUN2*+/+ (grey) and *NSUN2*−/− (red) cells in G0/G1- (K), S- (L), and G2/M- (M) phases of the cell cycle after treatment with sodium arsenite for the indicated time. (*n* = 3 samples per time point). Data presented as mean, error bars ± SD. *p*-Value: two-way ANOVA calculating row (grey; treatment) and column (black; genotype) factor variation. The underlying data for this figure can be found in S1 File. CHX, cycloheximide; eIF2, eukaryotic initiation factor 2; FC, fold-change; Norm. abs., normalised absorbance; OP-puro, O-propargyl-puromycin; wt, wild-type.

The dynamic regulation of protein synthesis is an integral part of the cellular stress response [34]. Global protein synthesis is repressed in response to oxidative stress, and translation of mRNAs that encode specific stress-related proteins is enhanced (Fig 3A). Therefore, we analysed how global protein synthesis changed over time in response to stress by measuring OP-puro incorporation (Fig 3B). In *NSUN2*+/+ cells, protein synthesis rates changed dynamically upon sodium arsenite (NaAsO_2_) treatment and recovered within 4 hours (Fig 3C; S3A Fig). NSUN2-depleted cells showed attenuated changes to protein synthesis rates (Fig 3C and 3D; S3A Fig), which we confirmed using fluorescence imaging (S3B and S3C Fig). Protein synthesis rates of rescued *NSUN2−*/*−* cells (NSUN2) were comparable to *NSUN2*+/+ cells and slightly, but not significantly, reduced when the enzymatic dead version of NSUN2 (K190M) was expressed (Fig 3E).

Polysome profiling confirmed a recovery of mRNA translation between 2 and 4 hours of stress (Fig 3F). We also measured a difference of the heavy polysomes after 4 hours in *NSUN2*+/+ cells, which appeared to be less evident in *NSUN2−*/*−* control or K190M-overexpressing cells (Fig 3F–3H; S3D Fig). *NSUN2−*/*−* cells rescued with the wild-type NSUN2 protein responded similarly to *NSUN2*+/+ cells and recovered the heavy polysomes after 4 hours of stress (Fig 3F and 3I; S3D Fig).

In summary, cell stress caused a strong but temporary reduction of protein synthesis, which was attenuated by loss of NSUN2.

### NSUN2 functions in the oxidative stress response by altering the cell cycle phases

To test for the functional relevance of NSUN2-regulated protein synthesis rates, we asked what impact NSUN2 deletion had on cell survival, as oxidative modifications to RNA can result in cell death [35]. In both NSUN2-expressing and -lacking cells, cell survival began to decrease between 2 and 4 hours after stress exposure, but at these early time points there were no differences in cell death (S3E–S3G Fig). We then asked whether cell division was affected by the absence of NSUN2, since the lengths of the cell cycle phases can play important roles in cellular adaptation and response to external stress stimuli [36]. For example, cells exposed to sodium arsenite often slow cell cycle progression to facilitate repair of oxidative lesions [37]. To test whether RNA methylation was required for adapting cell cycle to stress, we measured the cell cycle progression of NSUN2-expressing and -lacking cells upon exposure to arsenite (Fig 3J–3M; S3H Fig). In response to stress, the percentage of *NSUN2*+/+ cells decreased in the G1/G0-phase but increased in the S-phase and G2/M-phase of the cell cycle (Fig 3K–3M; grey). In contrast, the cell cycle progression of *NSUN2−*/*−* cells remained stable (Fig 3K–3M; red), indicating that *NSUN2−*/*−* cells failed to adapt the cell cycle phases to the stress stimulus. A tight regulation of global protein synthesis might be needed to avoid accumulation of proteins during cell cycle arrest and repair.

### Cytosine-5 RNA methylation is a metabolic sensor of external stress

**Fig 4:**
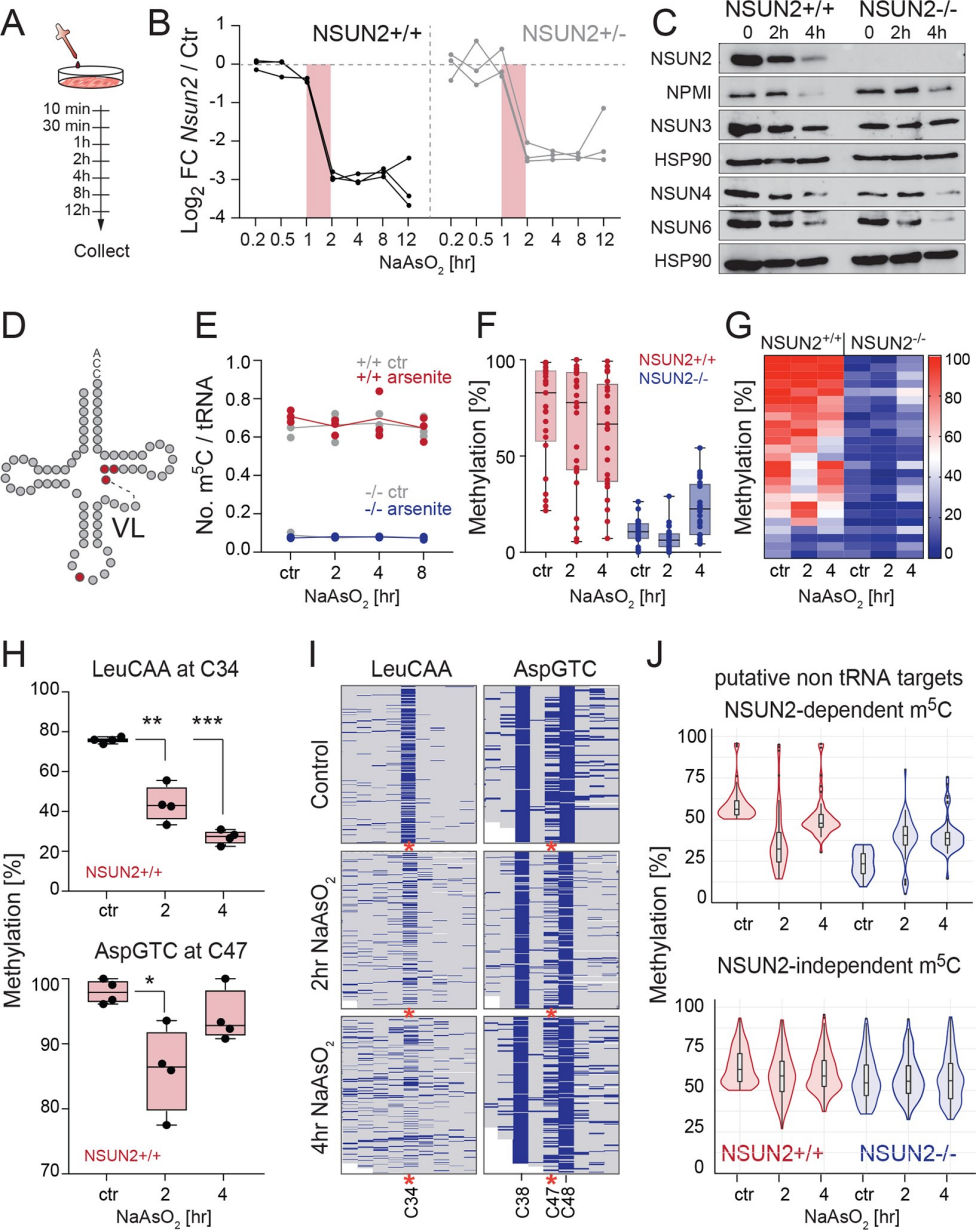
Levels of m^5^C changes site-specifically and dynamically in response to oxidative stress. (A) Time course of sodium arsenite treatment. (B) Log_2_ FC of *Nsun2* RNA expression in *NSUN2*+/+ and *NSUN2*+/− cells relative to GAPDH and normalised to the untreated control (‘Ctr’). Shown are 3 replicates. (C) Western blot analysis of the indicated proteins using whole cell lysates from *NSUN2*+/+ and *NSUN2*−/− cells. Hsp90 served as a loading control. (D,E) Detection of m^5^C in sodium arsenite–treated and untreated (‘ctr’) *NSUN2*+/+ and −/− cells using mass spectrometry. (*n* = 3 samples per time point). (F) Quantification of tRNA methylation percentage using RNA bisulfite sequencing of NSUN2+/+ and NSUN2−/− cells (*n* = 4 samples per time point). (G) Heatmap of methylation status of individual tRNA molecules shown in (F). (H,I) Quantification (H) and heatmap (I) of methylation changes in the tRNAs Leu^CAA^ and Asp^GTC^ in *NSUN2*+/+ and *NSUN2*−/− cells. (J) Quantification of methylation in non-tRNA targets. Data represent median in F, H, and J. Error bars are ±SD. *p*-Values: Student’s *t* test, *p < 0.05 and **p < 0.01. ***p < 0.001. The underlying data for this figure can be found in S8 and S9 Data and S1 File. FC, fold-change; GAPDH, glyceraldehyde 3-phosphate dehydrogenase; HSP90, heat shock protein 90; m^5^C, 5-methylcytosine; NPMI, nucleophosmin; tRNA, transfer RNA; VL, variable loop.

Since the dynamic regulation of protein synthesis was reduced when cells lacked NSUN2 and the down-regulation of global translation is integral to the cellular stress response, we next asked whether NSUN2 acted as a sensor of the external stress stimulus. We measured how endogenous NSUN2 expression was affected by oxidative stress induced by exposure to sodium arsenite (Fig 4A). NSUN2 expression decreased sharply on RNA and protein levels between 1 and 2 hours of sodium arsenite treatment (Fig 4B and 4C), which coincided with the formation of stress granules in the cytoplasm (S4A Fig). Similarly, one stimulus of UVB exposure was sufficient to rapidly reduce *Nsun2* RNA levels in human epidermal and dermal cells (S4B Fig), whereas vehicle control treatments did not affect NSUN2 expression (S4C Fig).

The nucleolus, where NSUN2 resides, can act as a stress sensor [38,39]. Nucleophosmin (NPMI) is a marker for nucleolar stress, and we observed a rapid, strong down-regulation of both NPMI and NSUN2 upon arsenite treatment (Fig 4C). Additional NSUN family members residing in the mitochondria (NSUN3, NSUN4) and cytoplasm (NSUN6) were similarly repressed in response to arsenite stress (Fig 4C). In conclusion, expression of NSUN2 and at least three of its m^5^C RNA methyltransferase family members were repressed in response to oxidative stress.

### Stress induces a site-specific and dynamic loss of m^5^C

We then asked how stress-related loss of NSUN2 altered tRNA methylation (Fig 4D). Indeed, MS quantifying total m^5^C in tRNAs confirmed that cells chronically lacking NSUN2 (*NSUN2−*/*−*) show low levels of m^5^C (Fig 4E) [11]. However, acute depletion of NSUN2 by oxidative stress exposure resulted in no significant differences in total m^5^C levels in tRNAs (Fig 4E). Because tRNAs are highly abundant and stable and commonly contain m^5^C, we considered the possibility that early site-specific changes might not be detectable by MS. Therefore, we performed RNA BS-seq to quantify m^5^C at single-nucleotide resolution in tRNAs (S4D Fig) [11,40].

RNA BS-seq revealed only a modest overall reduction of cytosine-5 tRNA methylation at NSUN2-dependent sites in two independent experiments (Fig 4F; S4E Fig; S8A and S8B Data). NSUN2-independent methylated sites were unaffected (S4F Fig). Furthermore, our data allowed a high-resolution inspection of specific sites that require NSUN2 activity, which showed dynamic and reproducible changes in response to stress (Fig 4G–4I; S4G–S4I Fig). For example, methylation at C34 in Leu^CAA^ decreased gradually with arsenite treatment (Fig 4H and 4I; S4H Fig). Methylation of Asp^GTC^ at position C47 only significantly changed at 2 hours of arsenite treatment, whereas nearby sites (C38, C48) remained unchanged (Fig 4H and 4I; S4I Fig). We observed a similar change in methylation levels when we pooled all other potentially non–tRNA targeted sites by NSUN2, whereas other m^5^C sites were unaffected by stress (Fig 4J; S9A and S9B Data). Together, our data revealed that NSUN2-mediated deposition of m^5^C at distinct sites in tRNAs changed dynamically in response to oxidative stress.

### Dynamic changes of site-specific m^5^C levels require NSUN2

**Fig 5:**
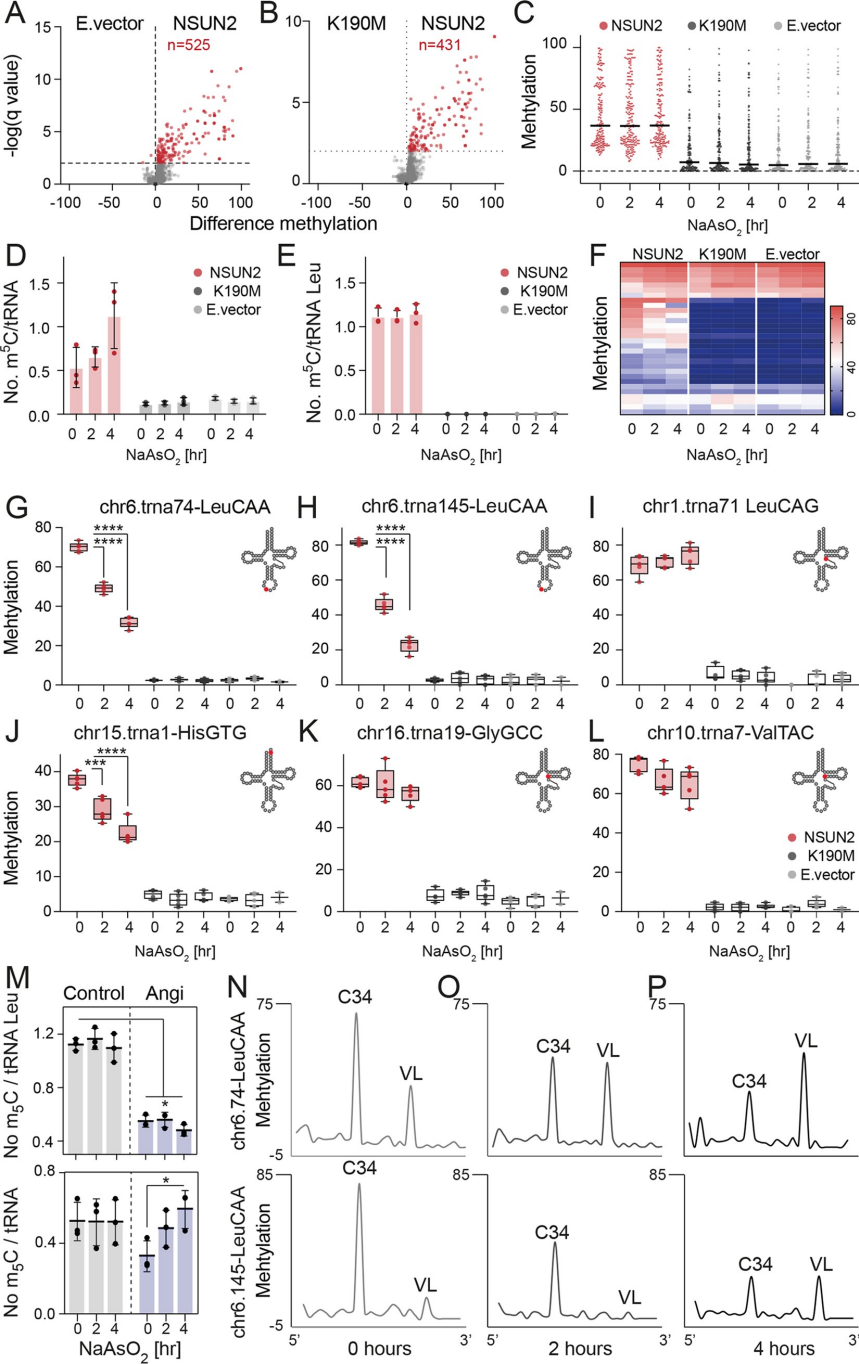
NSUN2-mediated tRNA methylation is dynamic and site-specific. (A, B) Volcano plot depicting the significant methylation changes when NSUN2 was reexpressed in *NSUN2*−/− cells compared to empty (‘E.’) vector (A) or K190M controls (B). (C) Global methylation levels of all m^5^C sites identified in (B) after treatment with arsenite for 0, 2, or 4 hours. Shown are all sites >20% methylation in NSUN2-rescued cells. (D, E) Mass spectrometry analyses to quantify the number of methylated sites (m^5^C) in all tRNA (D) or only tRNA Leu^CAA^ (E) in response to stress. (F) Heatmap showing all significantly different m^5^C sites (*p* < 0.05) changing upon stress in NSUN2-overexpressing cells. (G-L) Examples of m^5^C sites identified in (F). *padj < 0.05; **padj < 0.01; ****padj < 0.0001 (ordinary one-way ANOVA, multiple comparisons). (M) Mass spectrometry analyses to quantify m^5^C in tRNA leucine (upper panel) and all tRNAs (lower panel) in the presence of an angiogenin inhibitor (‘Angi’) and arsenite. Data presented as mean (*n* = 3), error bars ± SD. *p*-Value: *p*adj ANOVA. (N-P) Methylation levels (pooled from 5 replicates) of cytosines along tRNA 74-Leu CAA (upper panels) and 145-Leu CAA (lower panels) detecting all m^5^C sites within the tRNA molecule with different dynamic changes in response to stress. The underlying data for this figure can be found in S10 Data and S1 File. m^5^C, 5-methylcytosine; tRNA, transfer RNA; VL, variable loop.

Next, we asked whether NSUN2 was solely responsible for causing the site-specific methylation changes in tRNAs in response to oxidative stress. We rescued *NSUN2−*/*−* cells with NSUN2, K190M, or the empty vector as a control and performed RNA BS-seq. We selected for all sites with a minimum coverage of 100 and more than 5% methylation in the pooled replicates (*n* = 5) of untreated NSUN2-overexpressing cells (approximately 7,400 sites) (S10 Data). Reexpression of NSUN2 significantly restored 525 methylation sites when compared to the empty vector control (Fig 5A) and 431 sites when compared to K190M-overexpressing cells (Fig 5B). Reexpression of NSUN2 restored the level of tRNA methylation similarly to endogenous NSUN2 (S5A Fig).

We confirmed that the overexpressed proteins behaved like endogenous NSUN2 and were down-regulated upon exposure to arsenite (S5B Fig), and then we assessed how global methylation levels changed. We selected all significant sites with more than 20% methylation in the NSUN2-rescued cells when compared to K190M-infected cells and found that the overall median methylation levels remained largely unaltered (Fig 5C). MS for m^5^C using all tRNAs or tRNA Leu^CAA^ confirmed similar methylation levels after exposure stress (Fig 5D and 5E). Thus, global methylation levels in *NSUN2−*/*−* rescued cells remained largely stable in response to stress.

Then, we identified all individual m^5^C sites showing significantly different methylation levels in NSUN2-rescued cells after 2 or 4 hours of stress (Fig 5F). A majority of these specific sites required NSUN2 (Fig 5F), and their changes in methylation were highly comparable to cells expressing endogenous NSUN2 (*NSUN2*+/+; Fig 4G–4I). For instance, C34 in the anticodon loop of tRNA Leu^CAA^ exhibited up to 4-fold reduction of methylation (Fig 5G and 5H). Levels of m^5^C located to other positions showed a more modest reduction or were unaltered (Fig 5I–5L). Thus, we identified specific m^5^C sites that recapitulated the same changes in methylation when exposed to stress as described for *NSUN2*+/+ cells. Our rescue experiment therefore demonstrates that distinct m^5^C sites in tRNAs changed dynamically in response to stress and that these changes in methylation levels directly depended on NSUN2.

### Methylation levels within the same tRNA molecule are independent from each other

To explain why the site-specific methylation levels were not detected by MS, we first considered the possibility that unmethylated tRNAs escaped the MS analysis because of the long half-life of methylated tRNAs and the enhanced biogenesis of tRFs in response to stress. To test this hypothesis, we measured m^5^C in stress-exposed tRNAs in the presence of an angiogenin inhibitor (N65828) [41]. Indeed, we measured a reduction of m^5^C in all tRNAs and more than 2-fold reduction in tRNA Leu^CAA^ (Fig 5M), confirming that angiogenin was a major endonuclease cleaving unmethylated tRNAs (S5C Fig). However, the number of m^5^C per tRNA Leu^CAA^ decreased only slightly after 4 hours of stress (Fig 5M; upper panel). tRNA Leu^CAA^ contained two methylation sites, one in the anticodon C34 and one in the VL (Fig 5N–5P), and these sites were affected differently by oxidative stress. Only C34 decreased upon stress (Fig 5N–5P). We observed a similar dynamic change of methylation patterns within tRNA His^GTG^ (S5E Fig). Moreover, other leucine isotype tRNAs such as Leu^CAG^ were not methylated at C34 (S5D Fig). Thus, our data revealed that site-specific methylation of tRNAs changes dynamically during the cellular stress response even within the same tRNA molecule.

### Site-specific tRNA methylation determines tRFs biogenesis in response to oxidative stress

**Fig 6:**
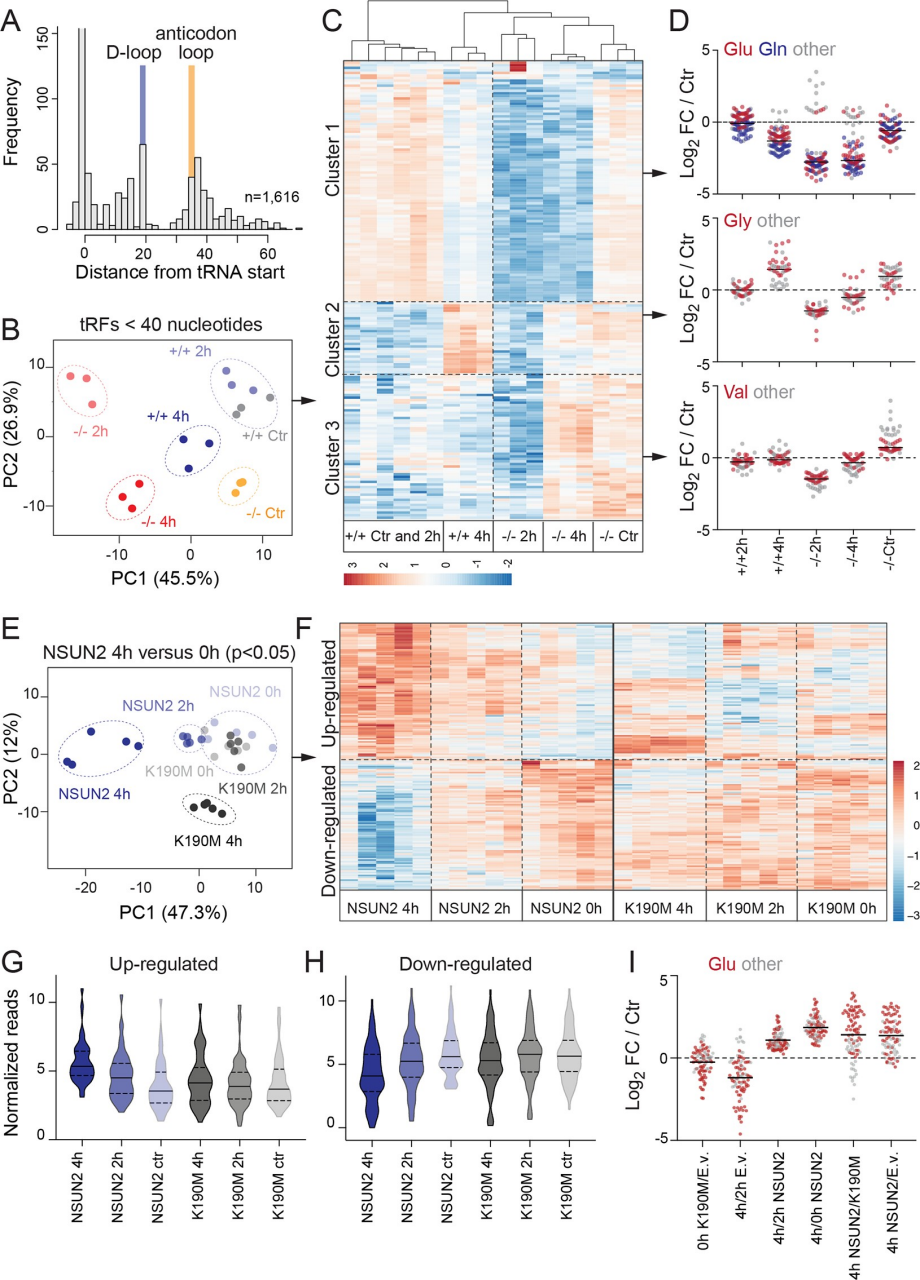
Site-specific tRNA methylation determines biogenesis of distinct tRFs. (A) Density plot of tRNA-derived sequences beginning at the indicated positions. (B) Clustering of tRNA-derived fragments < 40 nucleotides in *NSUN2*+/+ and *NSUN2−*/*−* cells untreated (‘Ctr’) or treated with sodium arsenite for 2 or 4 hours. Shown are 3 out of 4 replicates per time point. (C, D) Heatmap (C) and log_2_ FC of tRFs shown in (C). (E, F) PCAs (E) and heatmap (F) of significantly different tRFs in *NSUN2−*/*−* cells rescued with a wild-type (NSUN2) or point mutated (K190M) NSUN2 construct after 4 hours of exposure to stress compared to the untreated control (‘0h’). (G, H) Violin plots showing the read distribution of the tRFs shown in (E, F). (I) Log_2_ FC of the up-regulated tRFs when NSUN2-overexpressing cells are exposed to stress for 4 hours. tRNA glutamic acid–derived tRFs are highlighted in red. Line indicates the mean. The underlying data for this figure can be found in S11 and S12 Data and S1 File. FC, fold-change; PCA, principle component analysis; tRF, tRNA-derived fragment; tRNA, transfer RNA.

Next, we asked how altered expression of NSUN2 during the cellular stress response affected the biogenesis of tRFs. We performed small RNA-seq to identify all tRNA-derived sequences that significantly (*p*adj < 0.01) changed in *NSUN2−*/*−* cells after exposure to sodium arsenite for 2 hours (S11A and S11B Data). As expected, most tRNA-derived sequences started from the first tRNA nucleotide. Furthermore, we observed an enrichment of fragments starting at around positions 20 and 36, corresponding to the D-loop and the anticodon loop, respectively [42,43] (Fig 6A). Because some RNA modifications can stall reverse transcription (RT) and potentially cause sequencing biases, we only analysed tRNA-derived sequences with consistent coverage in all four replicates and omitted all fragments with missing read values in any of the conditions from the analyses (S11C Data).

Next, we focused specifically on tRFs that were smaller than 40 nucleotides, which capture the products of endonucleolytic cleavage (S11D Data). Principal component analysis (PCA) confirmed a clear separation by both genotype and treatment (Fig 6B), and tRFs from *NSUN2−*/*−* cells clustered closer to *NSUN2*+/+ cells exposed to stress for 4 hours (Fig 6B). Furthermore, tRFs clearly separated the 2- and 4-hours-treated *NSUN2−*/*−* cells from their untreated control. In contrast, *NSUN2*+/+ cells only showed distinct changes in tRFs when exposed for 4 hours to arsenite (Fig 6B). When subjected to stress, we discovered three distinct patterns of tRF production (Fig 6C; Cluster 1–3). Each cluster was dominated by distinct tRNA isoacceptors (Fig 6D), indicating that tRNAs were subjected to processing based on their sequence and potentially other corresponding internal modifications. Our data support a model in which all cells produce tRFs but the tRNA fragmentation pattern depends on the identity of the isoacceptor and the presence of modifications at distinct sites within the tRNA molecule.

In line with the reduction of NSUN2 protein expression in response to stress, tRFs derived from NSUN2-methylated tRNAs significantly increased after 4 hours of stress (S6A–S6C Fig; red and S5C Fig). The same tRFs remained unchanged or decreased in NSUN2-depleted cells (S6A–S6C Fig; blue and S5C Fig). In conclusion, the production of tRFs in response to stress differed substantially in the absence of NSUN2, confirming that tRF biogenesis was influenced by tRNA methylation, isoacceptor identity, and cellular stress.

### Biogenesis of tRF subsets is directly determined by tRNA methylation

To identify tRFs whose biogenesis directly required the enzymatic activity of NSUN2, we performed small RNA-seq analyses in the rescued *NSUN2−*/*−* cells that were either untreated or exposed to sodium arsenite. We identified all tRFs with differential abundance in NSUN2-rescued cells after 4 hours of stress (Fig 6E; S12 Data). Untreated NSUN2-rescued cells clustered close to K190M-overexpressing cells (Fig 6E), which was due to a distinct set of up-regulated and down-regulated tRFs (Fig 6F; ‘NSUN2 4h’). The production of these tRFs required NSUN2-mediated methylation because they remained unchanged in K190M-overexpressing cells (Fig 6G and 6H). As described for cells expressing endogenous levels of NSUN2 (*NSUN2*+/+), the up- and down-regulated groups of tRFs were dominated by specific tRNA isoacceptors (Fig 6I; S6D Fig). We concluded that NSUN2-mediated methylation at distinct sites within the tRNA molecule protected from processing into tRFs and thereby promoted efficient mRNA translation.

In particular, the formation of tRNA Glu^CTC^-derived tRFs depended on NSUN2 (Fig 6I). tRFs inhibit protein synthesis via several mechanisms including through direct inhibition of the ribosome or displacement of RNA-binding proteins [13,44,45]. To demonstrate that tRFs directly link stress sensing by NSUN2 to repression of protein synthesis, we independently added synthetic 5′ and 3′ tRNA Glu^CTC^ fragments to *NSUN2*+/+ and *−*/*−* cells and measured protein synthesis (S6E–S6H Fig). A scrambled noncoding RNA served as a negative control. Within 2 hours, 5′ and 3′ derived tRFs reduced protein synthesis in *NSUN2*+/+ cells (S6F Fig). Similar to the stress response, the protein synthesis rate was only temporarily repressed (S6F Fig). In contrast, protein synthesis in *NSUN2−*/*−* cells was unaffected by the introduction of additional tRFs, since *NSUN2−*/*−* cells are already saturated (S6G Fig; S6C Fig; S5C Fig). We confirmed that the transfection efficiency was comparable in *NSUN2*+/+ and *−*/*−* cells (S6H Fig). Thus, our data demonstrate that the intrinsic formation of tRFs is sufficient to dynamically regulate protein synthesis.

### m^5^C is required to balance anabolic and catabolic pathways during the stress response

**Fig 7:**
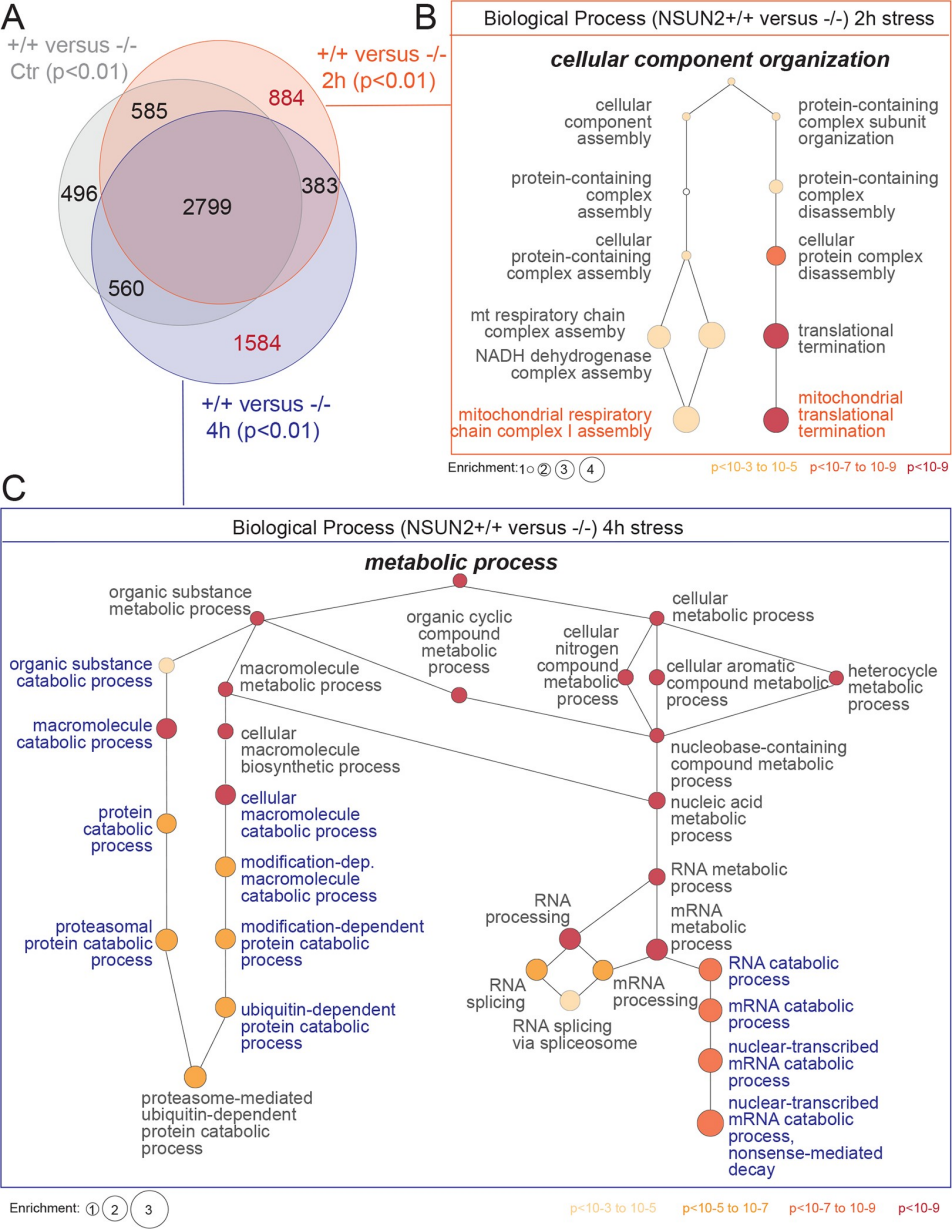
Loss of NSUN2 alters mitochondrial function and catabolic pathways in response to stress. (A) Venn diagram showing all significantly expressed genes in NSUN2*−*/*−* cells compared to NSUN2+/+ when untreated (‘Ctr’) or treated for 2 and 4 hours with sodium arsenite. (B, C) Gene enrichment analysis for biological processes (GO*rilla*) using the 884 uniquely changed genes in NSUN2*−*/*−* cells after 2 hours (B) or the 1,584 uniquely changed genes after 4 hours (C) of stress exposure. Colour code indicates *p*-value, and size reflects enrichment. The underlying data for this figure can be found in S13 and S14 Data.

To better understand how the failure to adapt protein synthesis rates to the external stress stimulus affected metabolic pathways, we identified all differentially expressed genes in *NSUN2−*/*−* cells after exposure to sodium arsenite (Fig 7A; S13A–S13C Data). A total of 2,799 genes were differentially expressed in *NSUN2−*/*−* cells when compared with *NSUN2*+/+ cells in all conditions and thus not influenced by the stress signal. We identified 884 and 1,584 misregulated genes in *NSUN2−*/*−* cells after 2 and 4 hours of stress, respectively (Fig 7A).

Two hours after stress exposure, regulators controlling mitochondrial function were significantly enriched in the differentially expressed genes (Fig 7B; S14A Data). In particular, genes encoding for complex I subunit assembly significantly changed in the absence of NSUN2. Complex I is the major entry point for electrons into the respiratory chain and likely to act as the rate-limiting step in respiration [46]. In line with these results, we confirmed that *NSUN2−*/*−* cells contained less-active mitochondria in particular during the early stress response (S7A and S7B Fig).

Four hours after stress exposure, the differentially expressed genes were significantly enriched in regulators of RNA and protein catabolic pathways (Fig 7C; S14B Data). These results closely parallel those of our metabolic assay that showed how loss of m^5^C captures cells in a catabolic state and affected mitochondrial function (Fig 1).

Finally, we asked whether factors promoting the stable catabolic state in *NSUN2−*/*−* cells were translationally regulated and could therefore be identified using ribosome profiling (Ribo seq). We used our previous published dataset (phs000645.v5.p1), in which we profiled *NSUN2−*/*−* cells that were rescued with NSUN2 or two different enzymatic dead versions of the protein (C271A, C321A) and compared them to the empty vector control cells (S15 Data) [12]. We confirmed that genes specifically enriched in the NSUN2-rescued cells were regulators of mRNA translation and RNA catabolic processes (S7C–S7E Fig). We now have further demonstrated that the formation of m^5^C balances protein synthesis with the metabolic requirements of stress responses.

In summary, our data revealed a highly dynamic regulation of protein synthesis rates in response to stress that is tightly coordinated by tRNA methylation and cleavage. We further discovered that loss of a major RNA methyltransferase shaped the biogenesis of tRNA fragments and thereby induced a catabolic cell state.

## Discussion

mRNA translation is a critical step for all gene expression programmes and also represents the most energy-consuming processes within cells [47]. Therefore, global and transcript-specific translation are continuously adapted to environmental cues including nutrients, growth factors, and stress stimuli. How precisely the external cues are sensed by the mRNA translation machinery, integrated into metabolic pathways, and then coordinated to trigger the appropriate cell response is less well understood. Here, we identify NSUN2 as an important sensor for oxidative stress. NSUN2 further links the environmental cue to the protein synthesis machinery via tRNA methylation. Loss of NSUN2 repressed global protein synthesis and thereby induced a catabolic cell state without affecting gene transcription.

External stress stimuli repress global protein synthesis, causing a switch of translation to stress- and cell type–specific regulatory proteins [48]. tRNAs play multiple regulatory roles in the adaptation of protein synthesis to the cellular stress response. For instance, the content of internal tRNA modifications and also the level of charged tRNAs rapidly changes in response to stress [49]. Loss of tRNA methylation at the VL causes global reduction of protein synthesis [11,12]. Changing the modifications at the wobble anticodon position alters transcript-specific translation [50–52].

Mature mammalian tRNAs are extremely stable, with an estimated half-life of 2–4 days [49,53], making the detection of dynamically changing m^5^C sites within hours of a stress response challenging. Nevertheless, we detected site-specific changes by RNA BS-seq. How m^5^C was site-specifically removed in distinct tRNAs is unknown, as no eraser protein has been identified so far. Alternatively, unmodified tRNAs might have a higher turnover and are therefore more difficult to capture than fully modified tRNAs.

Nucleotide modifications outside the anticodon loop are often linked to differential processing and cleavage of tRNAs [10,11,54,55]. Accordingly, NSUN2-expressing and -lacking cell populations differed in their production of tRFs under normal conditions and when exposed to cell stress. Depending on NSUN2-expression and stress, we observed distinct tRNA processing patterns. For instance, tRFs derived from tRNA Glu^CTC^ were specifically enriched in NSUN2-depleted cells and decreased in abundance with stress. Similarly, synthetic tRNA Glu^CTC^ fragments efficiently repressed protein synthesis, yet the effect was only short-lived. How the half-life of tRFs is regulated is currently unknown, but our data strongly indicate a dynamic turnover of the tRFs in response to stress. A high turnover of distinct tRFs can also explain the only temporal repression of global protein synthesis after stress exposure.

In summary, NSUN2-mediated formation of m^5^C is critical for integrating the cellular metabolic state with global protein synthesis and thereby triggers the appropriate cellular responses to external stress stimuli.

## Methods

### Ethics statement

The research including mice has been regulated under the Animals (Scientific Procedures) Act 1986 Amendment Regulations 2012 following ethical review and approval by the University of Cambridge Animal Welfare and Ethical Review Body (AWERB) under the terms of the United Kingdom Home Office licences PPL80/2231, PPL80/2619 and PPL_P36B3A804.

### Transgenic mice

The NSUN2 knockout mice (homozygous *Nsun2*^Gt[D014D11]Wrst^) were generated and genotyped as previously described [18].

### Cell sorting and analysis

Mouse keratinocytes from skin in telogen at postnatal day (P)49 of wild-type or NSUN2^−/−^ male mice were isolated as follows: whole mouse back skin was sterilised with 10% Betadine and 70% ethanol and washed in phosphate-buffered saline (PBS). The dermal side was floated on 0.25% trypsin without EDTA (Thermo Fisher Scientific) for 2 hours at 37°C. The epidermis was subsequently scraped from the dermis and disaggregated by gentle pipetting in low-calcium medium with 10% FCS and filtered through a 70 μm cell strainer. The cells were pelleted and resuspended in the following antibodies to cell surface markers in 2% bovine serum albumin (BSA): PE-conjugated anti-ITGA6 (1:500, clone GoH3, eBiosciences), Alexa Fluor 647-conjugated anti-CD34 (1:50, RAM34, eBiosciences), and goat anti-PCAD (1:50, R&D Systems). After incubation for 30 minutes at 4°C, cells were washed twice in PBS. For detection of PCAD, cells were incubated for 10 minutes at 4°C with anti-goat Alexa Fluor 488-congugated secondary antibody (1:500, Thermo Fisher Scientific). Cells were gated using forward versus side scatter to eliminate debris. Doublet discrimination was carried out using pulse width. The viable cells were then gated by their exclusion of sytox. Cells were sorted with a MoFlo high-speed sorter (Beckman Coulter) as follows: ITGA6^high^/CD34+ as hair follicle BG stem cells and ITGA6^low^/P-CAD^high^ as HG progenitor cells.

### Amplification of RNA from flow-sorted cells

Whole back skin of wild-type or NSUN2^−/−^ male mice at P49 was collected as skin in telogen and at P32–34 as skin in anagen. Hair follicle stages in telogen and anagen were verified by HE staining for each skin biopsy. RNA from flow-sorted cells was purified using Pure-Link RNA Micro Isolation Kit (Thermo Fisher Scientific). RNA was purified from 10^5^ to 3 × 10^5^ ITGA6^high^/CD34+ per biological replicate and 10^4^ to 5 × 10^4^ ITGA6^low^/PCAD^high^ per biological replicate. For each biological replicate, flow-sorted cells from 1 to 3 mice were pooled. Total RNA (75 pg; with an RIN of 8 or above) were amplified using an adapted version of the Kurimoto protocol [56]. The main changes to their published protocol included the use of an increased amount of Superscript III, primers, and dNTPs as well as a longer RT reaction. Samples were amplified for a total of 29 cycles, and each sample was split into 4 during this amplification process and combined at the end to avoid errors introduced by PCR being overrepresented in the final library. Low-DNA/RNA bind tips and tubes were used throughout the experiment.

### Gene expression arrays and analyses

RNA samples were amplified using the Genechip WT Plus kit (Thermo Fisher Scientific). Briefly, the RNA was converted into cDNA and amplified using a mix of primers to target polyA and non-polyA mRNAs generating biotin-labelled cRNA. The cRNA was then hybridised to the Gene ST array, stained, and scanned using the GeneTitan Instrument (Thermo Fisher Scientific). After purification, quality control, and quantity normalisation, the cRNAs of four samples per genotype and condition were hybridised to the Affymetrix MouseWG-6 v2.0 Expression BeadChip (Illumina). Hybridisation, washing, staining, and scanning were performed according to standard Illlumina protocols (Illumina Whole-Genome Gene Expression DirectHyb Assay). Microarray hybridisation, washing, and scanning were performed at the Cambridge Genomic Services at the Department of Pathology (University of Cambridge, Cambridge, UK). Differential expression analysis was done with the R limma package using default settings.

### In situ hybridisation via RNAscope

*Nsun2* RNA in mouse skin was labelled using the RNAscope in situ hybridisation technology [57]. Freshly cut paraffin-embedded tissue sections were first heated at 56°C for 1 hour, dewaxed, and dehydrated. Endogenous peroxidases were blocked, followed by target retrieval steps (antigen retrieval and protease digestion). The *Nsun2* probe (Cat.426721) was hybridised for 2 hours at 40°C, followed by the six-step amplification protocol. The signal was developed with DAB, and slides were counterstained with 6% Mayer’s Haematoxylin, further dehydrated, cleared in Xylene, and mounted in DPX.

### Cell lines and culture conditions

*NSUN2*+/+ human dermal fibroblasts were purchased from Invitrogen (C0135C; Thermo Fisher). *NSUN2*+/*−* and two lines of *NSUN2−*/*−* human dermal fibroblasts were established as previously described [7]. All cells were cultured in Minimum Essential Medium (MEM, 31095–052; Thermo Fisher) supplemented with 20% HyClone FBS (SV30180.03; GE Healthcare) and 1% Penicillin-Streptomycin (P0781; Sigma-Aldrich). For the rescue experiments, *NSUN2−*/*−* cells were stably infected with the wild-type or an enzymatic dead version of NSUN2 carrying a single point mutation (K190M) [30]. Empty vector (pBABE)–infected cells served as a control. All *NSUN2−*/*−* rescue cells were cultured in MEM (31095–052; Thermo Fisher) supplemented with 20% HyClone FBS (SV30180.03; GE Healthcare) and no antibiotics.

Cells were passaged using Trypsin-EDTA (0.25%, 25200072; Thermo Fisher) diluted in PBS to a 1:1 ratio and split as required. Cells were maintained at 37°C in a humidified incubator with a 5% CO_2_ atmosphere. Cells were grown on plastic dishes or flasks of tissue culture grade, depending on the experiment (Falcon; Corning and Nunc; Thermo Fisher).

To induce oxidative stress, cells at 80% confluency were incubated with fresh prewarmed media containing 200 μM sodium arsenite (stock solution 200 mM in PBS; NaAsO_2_, Sodium [meta]arsenite, S7400; Sigma-Aldrich) for the indicated time. To induce UV radiation stress, cells at 80%–90% confluency without medium were exposed to 100 J/m2 of UV light in a CL-1000 Ultraviolet Crosslinker (UVP). Fresh media were added directly after the exposure. To label the active mitochondria, the MitoTracker Red CMXRos reagent was used (M7512; Invitrogen) following the manual’s recommendations. Cells were incubated with 200 mM MitoTracker reagent (in DMSO) dissolved in cell culture medium for 30 minutes. To inhibit the mTOR pathway, rapamycin was used at a 500 nm or 1 μM concentration diluted in cell culture medium (stock solution: 2.74 mM in DMSO, R8781; Sigma-Aldrich). To inhibit angiogenin, cells were exposed to 43 μM of the small-molecule inhibitor N65828 (8-amino-5-[40-hydroxybiphenyl-4-yl azo] naphthalene-2-sulphonate) obtained from the National Cancer Institute (http://dtp.cancer.gov) (stock aliquots of 1 mg/mL: 1 mg powder in 100 μl DMSO and 900 μl PBS). Cells incubated with 0.5% (v/v) PBS or 0.5% (v/v) DMSO in cell culture medium served as controls.

For transfection with tRNA fragments, cells were grown in 6-well plates and additionally transfected with synthetic 5′ and 3′ tRNA fragments. For this, 10 μM of tRNA fragments were mixed with antibiotic-free medium and the DharmaFECT1 Transfection Reagent, by following manufacturer's recommendations (T-2001, Dharmacon; GE Healthcare). The tRNA fragments used were 5′-Glu-CTC tRNA-F synthetic sense (5′ UCC CUG GUG GUC UAG UGG UUA GGA UUC GGC GCU CUC) and 3′-Glu-CTC-tRNA-F synthetic sense (5′ CCG CCG CGG CCC GGG UUC GAU UCC CGG UCA GGG AA) (Thermo Fisher). Mock control samples with just the transfection reagent and 10 μM of fluorescently labelled control siRNA (Qiagen) served as negative controls. Samples were collected in a time course for up to 24 hours posttransfection.

### Metabolic analysis

For LC-MS/MS, *NSUN2*+/+, *NSUN2*+/*−*, and two biological replicates of *NSUN2−*/*−* cells were cultured in 150 mm round dishes and in five technical replicates each. In total, 8 to 10 million cells were collected per sample, pelleted, and flash-frozen in liquid nitrogen until further analysis. In addition, back skin from NSUN2*−*/*−* was collected from P26–P27 male mice in anagen. A total of 5 wild-type and 3 NSUN2*−*/*−* mice were used. Fat and connective tissue was scraped off, and the skin samples were snap frozen in liquid nitrogen. Each sample (70 mg) was homogenised on ice in methanol/chloroform using a tissue homogeniser (Polytron PT2500, Kinematica). To assess the metabolic content of these samples, LC-MS/MS was performed and optimised with internal standards, for the targeted analysis of aqueous metabolites including methionine and TCA cycle intermediates, nucleotides, and amino acids [58,59]. For NMR spectroscopy, skin samples from 4 wild-type and 3 NSUN2*−*/*−* mice were prepared as described before and processed as previously described [60].

### RNA isolation, RT, and RT-qPCR

Total RNA was prepared using Trizol reagent (Thermo Fisher Scientific) according to manufacturer's instructions. For further purification, total RNA was subjected to 30 minutes TURBO DNase treatment (AM2239; Invitrogen) at 37°C following manufacturer’s instructions. To inactivate the reaction, the samples were Phenol:Chloroform (77617; Sigma-Aldrich) extracted and precipitated using sodium acetate (pH 5.5) and 1–2 μl Glycoblue (AM9516; Ambion) overnight at −80°C. The RNA was washed with ethanol and resuspended. The concentration of each sample was assessed using a Qubit Fluorometer and the Qubit RNA HS Assay Kit (Q32855; Invitrogen) following the kit's instructions.

Double-stranded cDNA was synthesised from 1 μg of RNA and the Superscript III reverse transcriptase (18080085; Life Technologies) following manufacturer’s instructions using Random Primers (C1181; Promega). Each RT quantitative PCR (RT-qPCR) reaction was set up in MicroAmp Optical 96-well plates (N8010560) using 1 μl cDNA, 5 μl TaqMan Fast Universal PCR Master Mix (4366073), 3.5 μl RNAse-free water, and 0.5 μl of a predesigned *Nsun2* probe (Hs00214829_m1; Applied Biosystems). A human GAPDH probe was used for normalisation using the ΔCt method (4333764T; Applied Biosystems). RT-qPCR and data acquisition were conducted using the StepOnePlus Real-Time PCR System (Applied Biosystems).

### Protein extraction and western blotting

Cells were first rinsed with PBS and lysed in ice-cold RIPA buffer (50 mM Tris-HCL [pH 7.4], 1% NP-40, 150 mM NaCl, 0.1% SDS, 0.5% Sodium deoxycholate– 1 mL RIPA per T75 or 100 mm culture dish). RIPA was supplemented with cOmplete Mini EDTA-free Protease Inhibitor Cocktail tablets (11836170001, Roche), and cells were collected using a cell scraper. The lysates were centrifuged for 15 minutes at maximum speed in a precooled centrifuge at 4°C and their supernatant collected and kept on ice. The concentration of each protein sample was assessed using the Pierce BCA Protein Assay kit (23225; Thermo Fisher) according to the manufacturer's instructions and measured using a spectrophotometer and the Softmax software for protein quantification.

Cell protein lysates were mixed with NuPAGE LDS Sample Buffer (4X) (NP0007; Invitrogen) and run on 7.5% or 12.5% separating polyacrylamide gels. PageRuler Prestained Protein Ladder (26616; Thermo Fisher) was loaded as a size marker. All gels were run in 1x SDS Running Buffer (30 g Tris Base, 144 g Glycine, 10 g SDS, and H_2_O to 1 L; for 10x buffer) at 160V for approximately 1 hour, after which the proteins were transferred to a nitrocellulose or PVDF membrane (GE Healthcare) in 1x Transfer Buffer (30.3 g Tris Base, 144 g Glycine, and H_2_O to 1 L; for 10x buffer) containing 15% (v/v) methanol at 90 V, for 1.5 hours on ice. Membranes were blocked for a minimum of 1 hour at room temperature in 5% (w/v) nonfat milk or 5% (w/v) BSA (A4503-50G; Sigma-Aldrich) in 1x TBS and 0.1% Tween-20 (TBS-T) (48.4 g Tris Base, 160 g NaCl, and H_2_O to 1 L; for 20x TBS buffer at adjusted pH 7.6) and incubated with primary antibody in blocking solution overnight at 4°C. Each membrane was washed three times for 10 minutes in TBS-T prior to incubation with the appropriate horseradish peroxidase (HRP)-labelled secondary antibody (1:10,000) in TBS-T at room temperature for 1 hour (anti-mouse NA931 and anti-rabbit IgG HRP NA934; GE Healthcare–anti-goat IgG HRP sc-2020; Santa Cruz). Finally, the membranes were washed as before and the antibodies detected by using the Amersham ECL Prime Western Blotting Detection Reagent (RPN2232; GE Healthcare). The primary antibodies used were NSUN2 (1:1,000, 20854-1-AP, Proteintech), NSUN3 (1:500, GTX46175, GenTex), NSUN4 (1:500, ab101625, Abcam), NSUN6 (1:500, 17240-1-AP, Proteintech), NPMI (1:500, B0556, Sigma-Aldrich), and HSP90 (1:500, sc-13119, Santa Cruz).

### Immunofluorescence

Cells were grown directly on autoclaved glass coverslips placed in 12-well culture dishes. Cell culture and sodium arsenite or recombinant angiogenin treatments were carried out as described before. To fix the cells, the glass coverslips were first briefly rinsed with wash buffer comprising 0.1% Tween-20 in 1x PBS and incubated for 10 minutes at room temperature with paraformaldehyde (4% PFA in PBS; Santa Cruz). Cells were then washed three times with ice-cold wash buffer for a couple of minutes. To examine intracellular proteins, cells were permeabilised for 10 minutes with PBS containing 0.25% Triton X-100 at room temperature and washed again three times for 5 minutes in wash buffer. This step was omitted for the study of membrane proteins. To block nonspecific antibody binding, cells were incubated with blocking buffer comprising 1% BSA and 22.52 mg/mL glycine in PBS with 0.1% Tween-20 (PBST) for 1 hour. To detect specific proteins of interest, cells were then incubated with primary antibodies diluted in 1% BSA in PBST at 4°C overnight. The cells were then washed three times in wash buffer for 5 minutes each. To label the detected proteins, cells were incubated with the respective Alexa Fluor secondary antibodies diluted in 1% BSA in PBST for 1 hour at room temperature on a shaker and protected from light (1:1,000; Life Technologies). Cells were washed as before and their nuclei counterstained with DAPI (1:3,000 in PBS; Sigma) for 5 minutes. Finally, cells were rinsed with wash buffer and the glass coverslips mounted on slides with PBS:glycerol solution (1:1). The primary antibodies used were Angiogenin (1:500, PC317L, Calbiochem), eIF4A1 (1:200, sc-14211, Santa Cruz), and p-eIF2a (1:1,000, 9721S, Cell Signaling).

### Detection of m^5^C by MS

Human dermal fibroblasts were grown and treated with sodium arsenite as described above. NSUN2+/+ and *NSUN2−*/*−* cells were cultured in 100 mm dishes. Three replicates per condition and genotype were used for the experiments. Cell pellets were collected and lysed directly in Trizol for RNA extraction. To measure the m^5^C modifications, optimised MS analysis was performed [61]. Total RNA was loaded on a size-exclusion column (Agilent Bio SEC-3, 3 μm, 300 Å, 7.8 × 300 mm, Agilent, Waldbronn, Germany) and RNA fractions eluted with 100 mM ammonium acetate at pH 7 as the mobile phase. tRNA was separated from rRNA and small RNAs, vacuum concentrated, and reconstituted in water. tRNA concentrations were determined by UV spectroscopy at 260 nm. rRNA and other large RNA species were excluded from the fractions.

Purified tRNA (approximately 400 ng) was digested using a mixture of benzonase (2 U), bacterial alkaline phosphatase (2 U), and phosphodiesterase I (0.2 U) in a final reaction volume of 20 μL. The reaction mixture was supplemented with MgCl_2_ to a final concentration of 1 mM and Tris-HCl (pH 8.0) to a final concentration of 50 mM. Nucleobase deaminase inhibitor coformycin and tetrahydrouridine were added at a final concentration of 10 μg/mL and 50 μg/ml, respectively, and butylated hydroxytoluene (an antioxidant) was added at a final concentration of 0.5 mM (for further detail, see [62]). The digestion was allowed to proceed for 2 hours at 37°C and was stopped by filtering through a 10 kDa MWCO filter (AcroPrep Advance, 350 μl, Omega 10K MWCO, Pall, Dreieich, Germany) at 3,000*g* for 30 minutes. After addition of 10 μl pure water for salt dilution purposes, 18 μL of filtrate was mixed with 2 μL of internal standard (produced as recently described [63]). Each sample (10 μL) was injected for LC-MS/MS analysis (corresponding to around 150 ng tRNA digest). Calibration solutions for absolute quantification were prepared as recently described [63].

For quantification, an Agilent 1290 Infinity II equipped with a DAD combined with an Agilent Technologies G6470A Triple Quad system and electro-spray ionisation (ESI-MS, Agilent Jetstream) was used. Operating settings were as follows: positive ion mode, skimmer voltage 15 V, cell accelerator voltage 5 V, N_2_ gas temperature 230°C and N_2_ gas flow 6 L/minute, sheath gas (N_2_) temperature 400°C with a flow of 12 L/minute, capillary voltage of 2,500 V, nozzle voltage of 0 V, and the nebuliser at 40 psi. The instrument was operated in dynamic MRM mode, and the individual MS parameters for the nucleosides are summarised in S16 Data. The mobile phases were A as 5 mM NH_4_OAc (≥99%, HiPerSolv CHROMANORM, VWR) aqueous buffer, brought to pH 5.6 with glacial acetic acid (≥99%, HiPerSolv CHROMANORM, VWR), and B as pure acetonitrile (Roth, LC-MS grade, purity ≥ 99.95). A Synergi Fusion-RP column (Phenomenex, Torrance, CA, United States; Synergi 2.5 μm Fusion-RP 100 Å, 150 × 2.0 mm) at 35°C and a flow rate of 0.35 ml/minute were used. The gradient began with 100% A for 1 minute and increased to 10% B by 5 minutes and to 40% B by 7 minutes. The column was flushed with 40% B for 1 minute and returned to starting conditions to 100% A by 8.5 minutes followed by re-equilibration at 100% A for 2.5 additional minutes.

For the isolation of single tRNA isoacceptors, a modified approach was used to the one previously described in [64]. Briefly, tRNA LeuCAA was hybridised with a reverse complementary, biotinylated DNA-oligonucleotide (AGT CTG GCG CCT TAG ACC ACT CGG CCA TCC TGA CAA A [Biotin]) followed by immobilisation on streptavidin-coated magnetic beads (Dynabeads MyOne Streptavidin T1, Life Technologies). The hybridisation step was performed in 5× SSC buffer (20x: 3 M NaCl, 300 mM trisodium citrate [pH 7.0]) using 1 μl biotinylated oligonucleotide (100 μM) and 1 μg of SEC purified total tRNA per 25 μl beads. Samples were denatured at 90°C for 3 minutes, hybridised at 65°C for 10 minutes, and cooled to room temperature. The magnetic Dynabeads were washed as described in [64]. Immobilisation of the hybrid was performed at 25°C under shaking for 30 minutes. The supernatant containing nontarget tRNAs was removed, and the beads were washed once in 1× SSC buffer and three times in 0.1× SSC buffer. Finally, the beads were resuspended in 20 μl MilliQ water and heated to 80°C for 3 minutes to elute the target tRNA. No further DNase treatment was carried out. The isolated tRNA was digested with 10 μl digestion mix (final volume: 30 μL). Finally, 9 μl of the digested and isolated tRNA was coinjected with 1 μl stable isotope-labelled internal standards for measurement by MS [63].

### Sucrose density centrifugation

Sucrose gradients were used to separate subpolysomal and polysomal ribosomes. Sucrose gradients (10%–50% [w/v]) were prepared in gradient buffer (300 mM NaCl_2_, 15 mM MgCl_2_, 15 mM Tris-HCL [pH 7.5], 1 mM DTT, 0.1 mg/ml CHX).

Human dermal fibroblasts were grown and treated with sodium arsenite as described above. At 70%–80% confluency, cells were washed twice in PBS-CHX (100 μg/ml) and scraped into 500 μl lysis buffer (300 mM NaCl_2_, 15 mM MgCl_2_, 15 mM tris-HCL [pH 7.5], 1 mM DTT, 0.2 M sucrose, 0.1 mg/ml CHX, 0.5% IGEPAL, 100 U RNasin Plus RNase Inhibitor [N2615; Promega] per 0.5 ml). Cell lysates were incubated on ice for 3 minutes prior to pelleting the cells at 1,300*g* for 5 minutes. The supernatant was then layered on top of the gradient and centrifuged at 38,000 rpm (acceleration 9, deceleration 6) for 2 hours at 4°C using an ultracentrifuge (Beckman Coulter). Gradients were fractioned using a gradient fractionation system (Presearch), and fractions were collected at 1-minute intervals using the FOXY Jr collection system (Presearch) at a flow rate of 1 ml/minute. Absorbance was measured constantly at 254 nm using a UA-6 UV-VIS detector (Presearch).

### Sequencing library preparation

For all sequencing experiments, total RNA was isolated from human dermal fibroblasts, as described. The quality of the RNA and prepared libraries was assessed using the appropriate high-sensitivity RNA and DNA microfluidics chip on the Agilent Bioanalyzer platform (Agilent Technologies). All cDNA libraries were subjected to a cleanup step with the use of Agencourt AMPure XP beads (Beckman Coulter) and further multiplexed for sequencing on a HiSeq platform (Illumina).

### RNA BS-seq

RNA BS-seq libraries were generated from untreated and sodium arsenite–treated human dermal fibroblasts for 2 or 4 hours. Two independent experiments were performed; the first consisted of 4 and the second of 5 replicates per genotype and time point. Thus, a total of 9 replicates per condition and genotype were processed and analysed. To generate the samples, RNA was prepared and BS-treated as previously described [11]. Briefly, 15 μg of total RNA was depleted from the ribosomal RNA population by using the Ribo-zero kit (MRZH11124, Illumina). Approximately 1–2 μg of rRNA-depleted samples were then BS-treated by mixing them with 42.5 μl 40% sodium BS solution (pH 5.0) and 17.5 μl DNA protection buffer supplied with the EpiTect Bisulfite Kit (59104; Qiagen). The reaction mixture was then incubated for three to four cycles of 5 minutes at 70°C, followed by 1 hour at 60°C on a thermal cycler. To desalt the reaction, all samples were passaged through Micro Bio-Spin 6 chromatography columns, following the manufacturer's instructions (732–6221; Bio-Rad), and next desulfonated by adding an equal volume of 1 M Tris (pH 9.0) to the reaction mixture and incubating for 1 hour at 37°C. BS-treated RNA samples were then precipitated overnight with 2.5 volumes of 100% ethanol, 0.1 volumes of 3 M sodium acetate (pH 5.5), and 1–2 μl Glycoblue (AM9516; Ambion). To repair the 2′,3′-cyclic phosphate and 5'-hydroxyl termini produced during the BS/desulfonation reaction, T4 Polynucleotide Kinase (PNK) was used by mixing the RNA with 10x T4 PNK reaction buffer, 10 mM ATP, 10 U of T4 PNK enzyme, and RNase-free H_2_O to a final volume of 50 μl (M0201S; New England Biolabs). This was incubated at 37°C for 30 minutes, followed by a heat inactivation of the enzyme at 65°C for 20 minutes and an overnight precipitation of the RNA, as before. Approximately 400 ng of BS-converted RNA was finally used to generate BS-seq libraries. Taking into account that the above BS treatment and desulfonation protocol cleaves long RNAs into 100-nucleotide fragments, the TruSeq Small RNA preparation kit was used (Illumina) to generate platform-compatible libraries. In brief, kit-provided RNA-seq adapters were ligated to the BS-converted RNAs, reverse transcribed at 50°C for 1 hour with SuperScript III and 2 mM of each dNTP (18080085; Thermo Fisher Scientific), and followed by a PCR amplification programme, following the user manual's recommendations, to finalise the process.

### Standard and small RNA-seq

Standard RNA-seq libraries were generated from human dermal fibroblasts previously treated with sodium arsenite for 2 or 4 hours. All cell samples were processed in four replicates per condition. Total RNA was extracted as described and depleted from ribosomal RNA using the Ribo-zero rRNA Removal Kit (MRZH11124, Illumina). The rRNA-depleted RNA was then used to generate RNA-seq libraries using the NEXTflex Rapid Directional RNA-seq Kit (5138–08; Illumina) or RNAHyper with Riboerase Kit (NEB).

Small RNA libraries were generated from human dermal fibroblasts treated with sodium arsenite for 2 or 4 hours, as described. At least four replicates per condition and genotype were processed and analysed. To generate the small RNA libraries, 10 μg of total RNA was subjected to a deaminoacylation reaction by mixing the purified RNA with 0.1 M Tris-HCl (pH 9.0), 1 mM EDTA, and RNAse-free H_2_O to a final volume of 300 μl. This reaction was incubated for 30 minutes at 37°C to deaminoacylate mature tRNAs. To further size-select the RNA, samples were loaded on Novex TBE Urea 6% gels (EC68652BOX; Thermo Fisher) and the desired RNA fractions purified. TBE Urea gels were pre-run in 1x TBE buffer (for 5x stock: 54 g Tris base, 27.5 g boric acid, 20 mL of 0.5 M EDTA [pH 8.0]) at 200 V for 30 minutes, while each sample was mixed with 2x Denaturing Sample Buffer (LC6876; Thermo Fisher) and denatured at 80°C for 5 minutes. To avoid renaturation, all samples were kept on ice. The polyacrylamide gels were then loaded with the RNA samples and the RNA Marker Low Easy ladder (R0002; Abnova) and run at 180 V for 1 hour. Each gel was next stained with ethidium bromide in 1x TBE buffer on a shaker for a few minutes, prior to being visualised over a UV transluminator. Using a scalpel and the RNA ladder as a guide, 20–150 bp fragments were excised. The gel piece was placed in a 0.5 ml tube pierced with a needle to create perforations, fitted in a 2 ml RNAse-free tube. To shatter the gel, the tubes were centrifuged for 5 minutes at maximum speed in a precooled 4°C benchtop centrifuge, and the gel pieces were eluted in 600 μl NaCl and 1 μl RNasin Plus RNase Inhibitor (N2615; Promega) overnight while rotating in a cold room. To remove any gel remnants and salts, each gel eluate was passed through a sterile Costar Spin-X centrifuge tube filter (CLS8160; Corning). The RNA flow-through was precipitated overnight, with ethanol and sodium acetate in −80°C. To collect the size-selected RNA, all samples were centrifuged for 30 minutes at maximum speed in a precooled 4°C benchtop centrifuge, the pellets washed with 70% ethanol, air dried, and finally resuspended in 12 μl RNAse-free H_2_O. To prepare the ends of the fragments for adapter ligation and library preparation, the samples were first heated at 70°C for 10 minutes and then mixed with 10x T4 PNK Reaction Buffer, 20 U of T4 PNK enzyme, and RNase-free H_2_O to a final volume of 50 μl (M0201S; New England Biolabs). This was incubated at 37°C for 1 hour, followed by a heat inactivation of the enzyme at 65°C for 20 minutes and a final phenol:chloroform cleanup step and overnight ethanol precipitation. The final RNA pellet was resuspended in 7 μl RNAse-free H_2_O. The libraries were generated using the TruSeq Small RNA Preparation Kit (Illumina), for which 3′ adenylated and 5′ phosphorylated adapters, suitable for Illumina RNA-seq, were ligated to an average of 400 ng small purified RNA fractions. Samples were then reverse transcribed at 50°C for 1 hour with the SuperScript III cDNA synthesis kit (18080085; Thermo Fisher Scientific), followed by a PCR amplification programme with Phusion DNA polymerase (F530S; Thermo Fisher Scientific).

### Sequencing data analyses

For all sequencing datasets, automatically generated FastQC reports (http://www.bioinformatics.babraham.ac.uk/projects/fastqc) were used for the initial assessment of the quality and basic processing of the reads. Sequencing adapters were trimmed from the 5′ and 3′ ends of the reads using cutadapt (v.1.8.1; https://pypi.python.org/pypi/cutadapt/1.8.1).

For standard RNA-seq data, adapters were removed and paired-end RNA-seq reads were aligned to the human reference genome (GRCh38/g38) using Tophat2 (v.2.0.9; options:–read-mismatch 2 –max-multihits 1 –GTF) guided by ENSEMBL gene models (release 82), while allowing for two mismatches per read and unique alignments only. In order to determine the mRNA abundance, RNA-seq read counts for the full transcript were obtained using featureCounts (v1.5.0-p1; http://subread.sourceforge.net; options: -O—minOverlap 10 -p -C -M -B -t exon -g gene_id -T 6 -a). The datasets were then normalised, and the statistical significance of differential expression was evaluated by using the R/Bioconductor DESeq2 package (https://bioconductor.org/packages/release/bioc/html/DESeq2.html).

To determine RNA methylation levels using RNA BS-seq data, a tailored approach to the one previously published was followed [11]. BS-seq reads were aligned to the human reference genome (GRCh38/hg38) by using Bismark (http://www.bioinformatics.babraham.ac.uk/projects/bismark; v.0.14.4; options: ‘–directional–n 1 –l 100’). To complete the mapping, tRNA gene predictions were obtained from GtRNAdb (http://lowelab.ucsc.edu/GtRNAdb). Heatmaps displaying either C or T in the aligned reads at each cytosine position were generated using custom PERL scripts and matrix2png (http://www.chibi.ubc.ca/matrix2png/) for visualisation. Cytosine positions on the heatmaps were reported relative to the annotated transcriptional start sites of the documented beginning of each tRNA.

For small RNA-seq data and to determine the abundance of tRNA fragments, a tailored approach was followed [11] in which first adapter-trimmed paired-end reads were mapped to the human reference genome (GRCh38/hg38) using bowtie (v.2.1.0; http://bowtie-bio.sourceforge.net/bowtie2; options:—no-mixed—no-discordant—end-to-end), while considering only reads that mapped uniquely to the genome. To annotate the tRNA-seq fragments, tRNA genes were downloaded from GtRNAdb (http://lowelab.ucsc.edu/GtRNAdb). Counts per tRNA fragments were normalised, and their respective abundances were statistically evaluated using the R/Bioconductor DESeq2 package (https://bioconductor.org/packages/release/bioc/html/DESeq2.html) and represented as log2(DESeq2-normalised counts).

For tRNA fragment analyses, four replicates per condition and genotype were used from the second biological replicate. To identify tRNA-derived sequences, all significant fragments (*p* < 0.01) in NSUN2*−*/*−* cells versus NSUN2+/+ cells after 4 hours of sodium arsenite treatment were selected and filtered for fragments smaller than 40 nucleotides. All rows with missing values (0) in any condition were excluded. The PCAs and heatmaps show all four or the most similar three replicates. PCA and heatmaps were generated using https://biit.cs.ut.ee/clustvis/.

Gene set enrichment analyses were done using Enrichr (http://amp.pharm.mssm.edu/Enrichr/) [65,66]. To analyse the affected biological processes and cellular pathways in response to stress, we used the RNA-seq data obtained from human dermal fibroblasts and performed gene enrichment analyses using GOrilla (http://cbl-gorilla.cs.technion.ac.il/) [67]. As running mode, we used ‘two ranked lists of genes’ using the 2,799 commonly regulated as a background list.

### Cell cycle and apoptosis

For cell cycle analyses, human dermal fibroblasts untreated and treated with sodium arsenite were briefly rinsed with PBS and collected with Trypsin-EDTA (1:1 in PBS). Following two consecutive washes with cold PBS, the cells were fixed and resuspended in ice-cold 70% ethanol. All samples were kept at 4°C until further processing. The samples were centrifuged at 12,000*g* for 5 minutes and rinsed twice with PBS. All pellets were resuspended in 3 ml PBS containing DAPI dye (1:3,000) and incubated at room temperature for 30–45 minutes while protected from light. The fluorescence of each sample was measured on a flow cytometer at 450/50 405 nm. To analyse the data, DAPI fluorescence measurements were visualised by histogram and the cell cycle phases determined by the curves and x-axis values.

For cell viability analyses, human dermal fibroblasts untreated and treated with sodium arsenite were assayed with the FITC Annexin V kit following the manufacturer's recommendations (556419; BD Biosciences). Briefly, cells were rinsed with PBS and collected with Trypsin-EDTA (1:1 in PBS). The fluorescence of each sample was measured at 530/30 488 nm and 610/20 561 nm. To analyse the data, fluorescence measurements were visualised by scatterplot, while compensation and quadrants were set up based on control samples of unstained cells, cells only stained with FITC Annexin V (no PI), and cells stained with PI (no FITC Annexin V).

### Global mRNA translation analysis

To investigate global protein synthesis, human dermal fibroblasts untreated and treated with sodium arsenite, recombinant angiogenin, or tRNA fragments were complemented with OP-puro and further labelled as previously described [16]. Reconstituted OP-puro (50 μM; 10 mM reconstituted stock [pH 6.4]; Medchem Source) was added to each sample in culture medium precisely 1 hour prior to collection. In each assay, a sample not treated with OP-puro served as a negative control, and as a positive control, samples treated with 50 μg/mL CHX (100 mg/ml in DMSO, C4859; Sigma-Aldrich) for 15 minutes were used. Cells were rinsed with PBS and then collected with Trypsin-EDTA (1:1 in PBS). After pelleting the collected cells and washing them once with PBS, these were resuspended in 0.5 ml PFA (1% w/v in PBS; Santa Cruz) and kept for 15 minutes on ice in the dark. Following fixation, samples were washed in PBS and permeabilised in PBS supplemented with 3% FBS and 0.1% Saponin (47036; Sigma-Aldrich) for 5 minutes at room temperature. To conjugate OP-puro to a fluorochrome, an azide-alkyne cycloaddition was performed for 30 minutes at room temperature in the dark. For this, the Click-iT Cell Reaction Buffer Kit (C10269; Life Technologies) and 5 μM of Alexa Fluor 555-Azide (A20012; Life Technologies) were used. To remove excess reagents and reduce the background signal, the cells were washed twice in PBS supplemented with 3% FBS and 0.1% Saponin. Finally, all samples were resuspended in PBS containing DAPI (1:3,000) and kept at 4°C until further analysis. The fluorescence of each sample was measured at 450/50 405 nm for DAPI and 585/15 561 nm for OP-puro. To analyse the data, fluorescence measurements were visualised by histogram, and the raw fluorescence values were extracted.

To visualise polypeptide synthesis in cultured cells, human dermal fibroblasts were grown directly on autoclaved glass coverslips placed in 12-well culture plates. Cell culture, sodium arsenite treatments, and the incorporation of OP-puro were carried out as described. To fix the cells, the glass coverslips were first briefly rinsed with 1x PBS and incubated for 15 minutes at room temperature with PFA (4% PFA in PBS; Santa Cruz). To permeabilise the cells and additionally block unspecific antibody binding, all coverslips were first incubated with 0.4% Triton X-100 in PBS for 15 minutes and next with 3% BSA in PBS for 1 hour at room temperature. To conjugate OP-puro to a fluorochrome, an azide-alkyne cycloaddition was performed for 30 minutes at room temperature and in the dark. For this, the Click-iT Cell Reaction Buffer Kit (C10269; Life Technologies) and 5 μM of Alexa Fluor 555-Azide (A20012; Life Technologies) were used. To remove excess reagents and reduce the background signal, the cells were washed once with 3% BSA in PBS. Op-puro-labelled cells were counterstained for their nuclei by using Hoechst (working dilution 0.5 μg/mL) for 10 minutes and mounted on glass slides with a PBS:glycerol solution (1:1). Glass slides were stored in the dark at 4°C until imaging.

### Flow cytometry data acquisition and analysis

Flow cytometry analysis of all cell assays described was performed with the LSRFortessa Flow Cytometer (BD Biosciences). Data were analysed using the FlowJo software. All samples were gated using forward versus side scatter to eliminate debris.

### Image acquisition and analysis

All bright-field and immunofluorescence images were acquired using an Axio Imager 2 Upright Research Microscope (Zeiss) with ORCA-Flash4.0 digital camera (Hamamatsu) or AxioCam MRc colour digital camera (Zeiss). All images were initially processed using the ZEN Microscope and Imaging Software and later the Adobe Photoshop CS6 package. The CellProfiler software packages were used for image quantifications and analysis. Finally, all images and figures were arranged using Adobe Illustrator CS7.

### Data analysis and statistical tests

Data analysis and plotting were performed with the RStudio package and the GraphPad Prism 7 software. Statistical tests were performed with GraphPad Prism 7. If not stated otherwise, data are presented as mean values, and error bars represent standard deviation (SD).

## Supporting information

S1 Fig Metabolic profiles of NSUN2-expressing and -lacking cells. (A) Schematic representation of stem cell differentiation in the absence or presence of NSUN2. (B) Marker expression and cellular differences in hair follicles expressing or lacking NSUN2. (C) Gating used for flow cytometry sorting of BG stem cells (ITGA6^high^/CD34^+^) in telogen (P49) wild-type and NSUN2*−*/*−* mice. (D) Gating used for flow cytometry sorting of HG progenitor cells (ITGA6^low^/PCAD^+^) in telogen (P49) wild-type and NSun2*−*/*−* mice. (E,F) Log2 FC (E) and Gene Ontology categories (F) of combined differential expressed genes (FDR < 0.05) in anagen and progenitor (ITGA6^low^/PCAD^+^) populations from skin of NSUN2+/+ and *−*/*−* mice. (G) Schematic representation of NSUN-dependent methylation at cytosine-5. (H) Overview of the one-carbon metabolism network. (I-N) Metabolic differences between NSUN2+/+ and NSUN2*−*/*−* mice relating to the methionine cycle (I,L), free amino acids (J,M), and free nucleotides (K,N) measured by NMR-based (I-K) or MS-based (L-N) metabolic profiling (*n* = 3–5 mice). (O) Model of how protein homeostasis changes the balance between protein synthesis and degradation in NSUN+/+ (upper panel) and NSUN2*−*/*−* (lower panel) cells. The underlying data for this figure can be found in S2 Data and S1 File. BG, bulge; DP, dermal papilla; FC, fold-change; FDR, false discovery rate; HG, hair germ; IFE, interfollicular epidermis; ITGA6, integrin alpha-6; MS, mass spectrometry; NMR, nuclear magnetic resonance; PCAD, P-cadherin; SAH, S-adenosyl-homocysteine; SAM, S-adenosyl-methionine; SG, sebaceous gland.
(TIF)

S2 Fig Rescue for loss of NSUN2 by reexpressing the wild-type or enzymatic dead protein. (A, B) Differentially expressed genes in *NSUN2−*/*−* compared to *NSUN2*+/+ cells (A) and *Nsun2* RNA levels in *NSUN2*+/+, +/*−*, and *−*/*−* cells (B) measured by RNA sequencing. (C, D) The transcriptional profile of *NSUN2−*/*−* cells overexpressing the NSUN2 protein is largely unaltered (C) although *Nsun2* is highly expressed (D). Expression of the empty (‘e.’) vector served as a control. (E) Venn diagram of differentially expressed genes (*p*adj < 0.01) in *NSUN2−*/*−* versus +/+ compared to NSUN2-rescued cells. (F) Two out of three replicates of polysome profiles using *NSUN2*+/+ and *−*/*−* cells. (G) Schematic representation of OP-puro incorporation in actively translating ribosomes. OP-puro mimics an amino-acyl-loaded tRNA molecule. (H) Example raw data outputs from OP-puro fluorescence analysis using a flow cytometer. CHX served as a control. (I) Protein synthesis measured by OP-puro incorporation in *NSUN2*+/+ and *−*/*−* cells after incubation with an angiogenin inhibitor (ANGi). (J) Western blot for NSUN2 and tubulin after incubation with 500 or 1,000 nm RAPA for 12 or 24 hours (h). (K) Quantification of protein expression shown in (J). (L) De novo protein synthesis in *NSUN2*+/+ and *−*/*−* after incubation with RAPA or CHX. DMSO served as a vehicle control (J-L). (M, N) Metabolic differences of *NSUN2−*/*−* cells rescued with the empty vector (‘e.v.’), K190M, or the NSUN2 protein shown as a PCA plot (M) or as Log_2_ FC differences of the significant different (*p* < 0.01 NSUN2 versus e.v.) metabolites (N). The underlying data for this figure can be found in S4 and S7 Data and S1 File. CHX, cycloheximide; OP-puro, O-propargyl-puromycin; PCA, principle component analysis; RAPA, rapamycin; tRNA, transfer RNA.
(TIF)

S3 Fig NSUN2 regulates cell cycle phases and global protein synthesis during the cellular stress response. (A) Example raw data outputs from OP-puro fluorescence analysis using a flow cytometer for human dermal fibroblasts treated with sodium arsenite. Dotted line represents the mean level of OP-puro positive control. (B) Immunofluorescence detection of OP-puro incorporation in human dermal fibroblasts. DAPI: nuclear counterstain. Scale bar: 20 μm. (C) Measurement of OP-puro fluorescence intensity in cells using microscope-acquired images. Each dot represents one cell. Data are represented as median. (D) Second replicate of polysome profiling of *NSUN2*+/+ and *NSUN2−*/*−* cells rescued with wt or mutated NSUN2 (K190M). The empty vector (‘e.V.’)-infected cells served as control (see Fig 3F–3I). (E) Example of raw data output from AnV and PI analysis to measure cell death. (F, G) Percentage of cells that are viable, apoptotic, or necrotic in *NSUN2*+/+ and *NSUN2−*/*−* cells exposed to sodium arsenite for the indicated hours (hr) (*n* = 3 samples per time point). (H) Summary of cell cycle distribution shown in Fig 3A–3D. Data represented as mean in (K-H). Error bars are ±SD. The underlying data for this figure can be found in S1 File. AnV, AnnexinV; OP-puro, O-propargyl-puromycin; PI, propidium iodide; wt, wild-type.
(TIF)

S4 Fig RNA methylation levels change dynamically in response to oxidative stress. (A) Immunofluorescence detection of the stress granules markers eIF4A1 (upper panels) and p-eIF2A (lower panels) in untreated (control) or sodium arsenite–treated *NSUN2*+/+ and *NSUN2−*/*−* cells. DAPI: nuclear counterstain. Scale, 20 μm. (B) *Nsun2* RNA levels in response to UVB exposure in primary human keratinocytes and dermal fibroblasts. (C) Western blot for NSUN2 in *NSUN2*+/+ and *−*/*−* cells incubated with vehicle control (DMSO, PBS). (D) Experimental outline of sample collection and RNA BS sequencing. (E,F) Quantification of tRNA methylation percentage of NSUN2-dependent (E) and -independent (F) sites in a second independent experiment (*n* = 5 samples per time point). (G) Second independent RNA BS-seq data shown as heatmap of methylation status of individual tRNA molecules in *NSUN2*+/+ and *NSUN2−*/*−* cells. (H, I) Quantification of methylation changes in the tRNAs Leu^CAA^ and Asp^GTC^ in *NSUN2*+/+ and *NSUN2−*/*−* cells shown in (E). Data represented as median in (E, F, H, I). One-way ANOVA adjusted *p*-value (H,I), **p < 0.005, ***p < 0.0005, ****p < 0.0001. The underlying data for this figure can be found in S8 Data and S1 File. BS, bisulfite; BS-seq, BS sequencing; eIF2, eukaryotic Initiation Factor 2; tRNA, transfer RNA.
(TIF)

S5 Fig Dynamic levels of NSUN2, tRFs, and methylation in response to stress. (A) Reexpression of NSUN2 but not K190M in *NSUN2−*/*−* cells restores methylation to similar levels of endogenous NSUN2 (*NSUN2*+/+). (B) Western blot for NSUN2 in *NSUN2−*/*−* cells infected with an empty (‘E.’) vector control, the enzymatic dead K190M, or the wild-type NSUN2 construct in cells untreated (0) or treated for 2 and 4 hours (h) with sodium arsenite. HSP90 served as a loading control. (C) Raw data (reads) for the indicated tRNAs obtained from small RNA sequencing in NSUN2-expressing (+/+; black) or -lacking (*−*/*−*; red) untreated (0h) or (h) treated 2 and 4 hours with arsenite. (D, E) Methylation levels (pooled from 5 replicates) of cytosines along tRNA 71-Leu CAG (C) and 1-His GTG (F) detecting m^5^C sites, in the variable loop (D) and C70 (E). The underlying data for this figure can be found in S10 Data and S1 File. HSP90, heat shock protein 90; m^5^C, 5-methylcytosine; tRF, tRNA-derived fragment; tRNA, transfer RNA.
(TIF)

S6 Fig Site-specific methylation shapes tRF formation to regulate protein synthesis. (A-C) Comparison of site-specific tRNA methylation and fragmentation in *NSUN2*+/+ (red) and *NSUN2−*/*−* (blue) cells. (*n* = 4 samples per time point). Data represent median and range. Adjusted *p*-value: one-way ANOVA. **p < 0.005, ***p = 0.0005. (D) Log2 FC of the down-regulated tRFs when NSUN2-overexpressing cells are exposed to stress for 4 hours. tRNA lysine-derived tRFs are highlighted in red; tRNA histidine-derived tRFs are highlighted in blue. Line indicates the mean. (E) Treatment regime to measure global protein synthesis of *NSUN2*+/+ and *−*/*−* cells transfected with tRNA Glu^CTC^-derived 5′ and 3′ tRFs after exposure to sodium arsenite. (F, G) Log_2_ FC of protein synthesis in *NSUN2*+/+ (F) and *NSUN2−*/*−* (G) in response to synthetic 5′ or 3′ tRFs. (*n* = 3 samples per time point). (H) A fluorescence siRNA was used as a control for transfection efficiency. The underlying data for this figure can be found in S11 and S12 Data and the S1 File. FC, fold-change; siRNA, small interfering RNA; tRF, tRNA-derived fragment; tRNA, transfer RNA.
(TIF)

S7 Fig Mitochondrial activity is reduced and catabolic pathways enhanced in the absence of NSUN2. (A, B) Mitochondrial activity (‘mito’) and protein synthesis (‘OP-puro’) after exposure to arsenite for the indicated time (hours) or CHX in *NSUN2*+/+ (A) and *NSUN2−*/*−* (B) cells. (C) GO analyses using Ribo seq data in *NSUN2−*/*−* cells rescued with NSUN2 or the enzymatic dead versions of NSUN2 C321A (left panel) and C271A (right panel). (D, E) PCA plot (D) and heatmap (E) of genes belonging to the GO: nuclear-transcribed mRNA catabolic process, nonsense-mediated decay. The underlying data for this figure can be found in S15 Data and S1 File. CHX, cycloheximide; GO, Gene Ontology; OP-puro, O-propargyl-puromycin; PCA, principle component analysis; seq, sequencing.
(TIF)

S1 Data Mouse skin microarray complete dataset. (XLSX)

S2 Data Mass spectrometry raw data. S2A: Mass spectrometry data for human dermal fibroblasts; S2B: Mass spectrometry data for mouse skin; S2C: NMR data for mouse skin. NMR, nuclear magnetic resonance.
(XLSX)

S3 Data Multiple *t* test for metabolites shown in Fig 1I–1K. (XLSX)

S4 Data RNA sequencing data. S4A: RNA sequencing data of *NSUN2*+/+, *NSUN2*+/*−*, and two lines of *NSUN2−*/*−* human dermal fibroblasts. S4B: RNA sequencing data *NSUN2−*/*−* cells reexpressing the NSUN2 protein or the empty vector as a control.
(XLSX)

S5 Data RNA bisulfite sequencing of *NSUN2−*/*−* cells rescued with the wild-type NSUN2 construct or the enzymatic dead NSUN2 construct K190M, and the empty vector as a control. (XLSX)

S6 Data Small RNA sequencing of rescued *NSUN2−*/*−* cells using the wild-type or enzymatic dead version of NSUN2 or the empty vector as control. (XLSX)

S7 Data Mass spectrometry data for rescued *NSUN2−*/*−* cells using the wild-type or enzymatic dead version of NSUN2 or the empty vector as control. (XLSX)

S8 Data RNA BS sequencing data. S8A: BS-seq tRNA methylated sites (*n* = 4 conversion assays) from first experimental replicate. S8B: BS-seq tRNA methylated sites (*n* = 5 conversion assays) from second independent experimental replicate. BS, bisulfite; BS-seq, BS sequencing; tRNA, transfer RNA.
(XLSX)

S9 Data RNA BS sequencing data identifying other potentially non–tRNA targeted sites by NSUN2. S9A: BS-seq to detect other potentially non–tRNA targeted sites by NSUN2 from 1 replicate (*n* = 4 conversion assays). S9B: BS-seq to detect other all sites not targeted by NSUN2 from 1 replicate (*n* = 4 conversion assays). BS, bisulfite; tRNA, transfer RNA.
(XLSX)

S10 Data RNA BS sequencing data of unstressed and stressed *NSUN2−*/*−* cells infected with the empty vector control, the wild-type NSUN2, or the enzymatic dead construct K190M. BS, bisulfite.
(XLSX)

S11 Data Small RNA sequencing in unstressed and stressed cells. S11A: tRNA fragments found in the small RNA-seq dataset. S11B: tRNA fragment sequencing and analysis (*p* < 0.01 *NSUN2−*/*−* at 2 hours of stress). S11C: tRNA fragment sequencing and analysis (samples with missing values removed). S11D: tRNA fragment sequencing and analysis (fragments smaller than 40 nucleotides). RNA-seq, RNA sequencing; tRNA, transfer RNA.
(XLSX)

S12 Data tRNA fragments in NSUN2, K190M, or empty vector infected cells after 0, 2, and 4 hours of treatment with sodium arsenite. (XLSX)

S13 Data RNA sequencing data. S13A: RNA-seq data from human dermal fibroblasts untreated (‘ctr’) or treated with sodium arsenite for 2 or 4 hours. Shown are transcriptional differences between *NSUN2−*/*−* and *NSUN2* +/+ cells in the control. S13B: RNA-seq data from human dermal fibroblasts untreated (‘ctr’) or treated with sodium arsenite for 2 or 4 hours. Shown are transcriptional differences between *NSUN2−*/*−* and *NSUN2* +/+ cells after 2 hours of stress. S13C: RNA-seq data from human dermal fibroblasts untreated (‘ctr’) or treated with sodium arsenite for 2 or 4 hours. Shown are transcriptional differences between NSUN2*−*/*−* and NSUN2 +/+ cells after 4 hours of stress. RNA-seq, RNA sequencing.
(XLSX)

S14 Data Gene ontology analyses. S14A: Gene enrichment (Cellular processes_Gorilla) for differentially expressed genes in *NSUN2*+/+ versus *NSUN2−*/*−* cells after 2 hours of exposure to sodium arsenite. S14B: Gene enrichment (Cellular processes_Gorilla) for differentially expressed genes in *NSUN2*+/+ versus *NSUN2−*/*−* cells after 4 hours of exposure to sodium arsenite. S14C: Gene enrichment (Cellular processes_Gorilla) for differentially expressed genes in *NSUN2*+/+ versus *NSUN2−*/*−* cells in untreated condition revealed no significant enrichment.
(XLSX)

S15 Data Ribo seq data of *NSUN2−*/*−* cells rescued with NSUN2 (wt) or enzymatic dead versions of the NSUN2 protein (C321C, C271A) or the empty vector (‘empty’) as control. wt, wild type.
(XLSX)

S16 Data Individual mass spectrometric parameters for the nucleosides. (XLSX)

S1 File Underlying data for Figs 1C–1E, 1I–1K, 2B–2D, 2D–2N, 3C–3E, 3K–3M, 4E, 4F, 4H, 4J, 5A–5E, 5G–5P, 6D and 6G–6I and S1E, S1F, S1I–S1N, S2B, S2D, S2I–S2L, S2N, S3C, S3F–S3H, S4B, S4E, S4F, S4H, S4I, S5A, S5D, S5E, S6A–S6D, S6F–S6H, S7A–S7C and S7E Figs. (XLSX)

## Abbreviations

ADMA: asymmetric dimethylarginine
BG: bulge
BS: bisulfite
BS-seq: BS sequencing
CHX: cycloheximide
FC: fold-change
FDR: false discovery rate
HB: hair bulb
HG: hair germ
IFE: interfollicular epidermis
ITGA6: integrin alpha-6; m^5^C, 5-methylcytosine
MS: mass spectrometry
mTORC1: mammalian target of rapamycin complex 1
NMR: nuclear magnetic resonance
NPMI: nucleophosmin
OP-puro: O-propargyl-puromycin
PCA: principal component analysis
PCAD: P-cadherin
RNA-seq: RNA sequencing
RT: reverse transcription
SAH: S-adenosyl-homocysteine
SAM: S-adenosyl-methionine
SDMA: symmetric dimethylarginine
TCA: tricarboxylic acid
tRF: tRNA-derived fragment
tRNA: transfer RNA
VL: variable loop
