# Supplementary figures and images for: Cytosine-5 RNA methylation links protein synthesis to cell metabolism
Source: PLoS Biol. 2019 Jun 14;17(6):e3000297. doi: 10.1371/journal.pbio.3000297 (PMC6594628; doi:10.1371/journal.pbio.3000297)

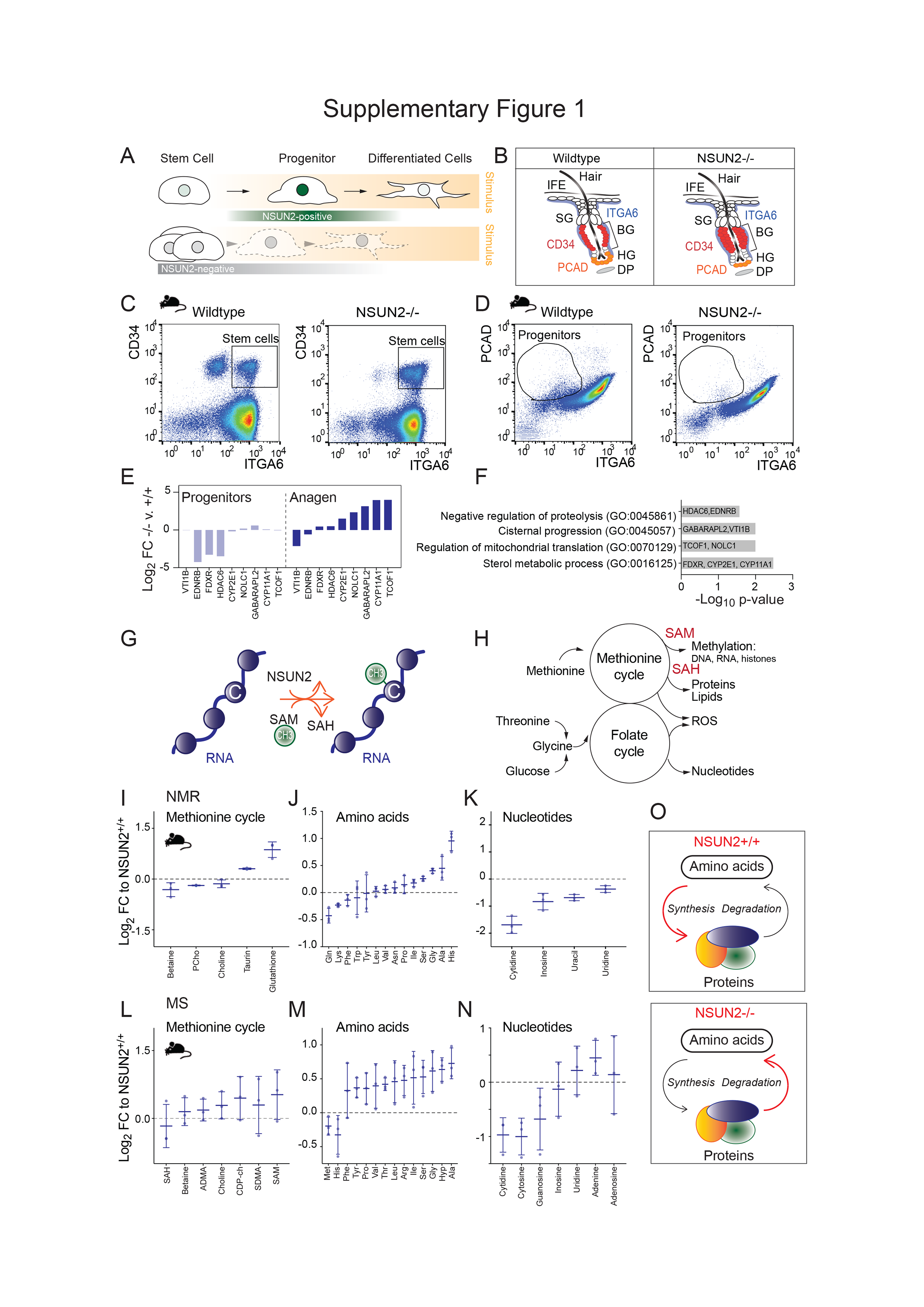

Supplement: S1 Fig — (A) Schematic representation of stem cell differentiation in the absence or presence of NSUN2. (B) Marker expression and cellular differences in hair follicles expressing or lacking NSUN2. (C) Gating used for flow cytometry sorting of BG stem cells (ITGA6high/CD34+) in telogen (P49) wild-type and NSUN2−/− mice. (D) Gating used for flow cytometry sorting of HG progenitor cells (ITGA6low/PCAD+) in telogen (P49) wild-type and NSun2−/− mice. (E,F) Log2 FC (E) and Gene Ontology categories (F) of combined differential expressed genes (FDR < 0.05) in anagen and progenitor (ITGA6low/PCAD+) populations from skin of NSUN2+/+ and −/− mice. (G) Schematic representation of NSUN-dependent methylation at cytosine-5. (H) Overview of the one-carbon metabolism network. (I-N) Metabolic differences between NSUN2+/+ and NSUN2−/− mice relating to the methionine cycle (I,L), free amino acids (J,M), and free nucleotides (K,N) measured by NMR-based (I-K) or MS-based (L-N) metabolic profiling (n = 3–5 mice). (O) Model of how protein homeostasis changes the balance between protein synthesis and degradation in NSUN+/+ (upper panel) and NSUN2−/− (lower panel) cells. The underlying data for this figure can be found in S2 Data and S1 File. BG, bulge; DP, dermal papilla; FC, fold-change; FDR, false discovery rate; HG, hair germ; IFE, interfollicular epidermis; ITGA6, integrin alpha-6; MS, mass spectrometry; NMR, nuclear magnetic resonance; PCAD, P-cadherin; SAH, S-adenosyl-homocysteine; SAM, S-adenosyl-methionine; SG, sebaceous gland. (TIF) [file pbio.3000297.s001.tif]

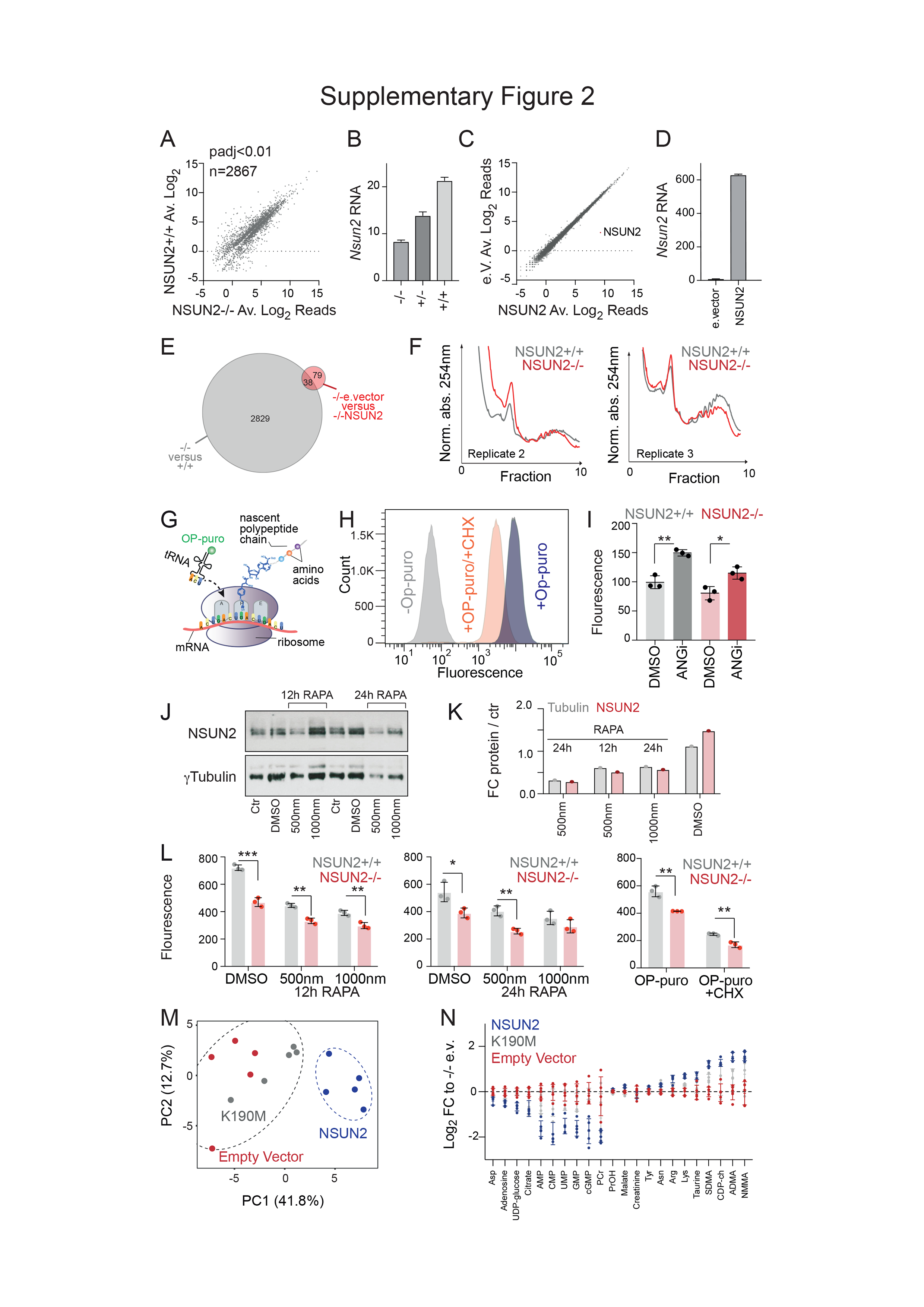

Supplement: S2 Fig — (A, B) Differentially expressed genes in NSUN2−/− compared to NSUN2+/+ cells (A) and Nsun2 RNA levels in NSUN2+/+, +/−, and −/− cells (B) measured by RNA sequencing. (C, D) The transcriptional profile of NSUN2−/− cells overexpressing the NSUN2 protein is largely unaltered (C) although Nsun2 is highly expressed (D). Expression of the empty (‘e.’) vector served as a control. (E) Venn diagram of differentially expressed genes (padj < 0.01) in NSUN2−/− versus +/+ compared to NSUN2-rescued cells. (F) Two out of three replicates of polysome profiles using NSUN2+/+ and −/− cells. (G) Schematic representation of OP-puro incorporation in actively translating ribosomes. OP-puro mimics an amino-acyl-loaded tRNA molecule. (H) Example raw data outputs from OP-puro fluorescence analysis using a flow cytometer. CHX served as a control. (I) Protein synthesis measured by OP-puro incorporation in NSUN2+/+ and −/− cells after incubation with an angiogenin inhibitor (ANGi). (J) Western blot for NSUN2 and tubulin after incubation with 500 or 1,000 nm RAPA for 12 or 24 hours (h). (K) Quantification of protein expression shown in (J). (L) De novo protein synthesis in NSUN2+/+ and −/− after incubation with RAPA or CHX. DMSO served as a vehicle control (J-L). (M, N) Metabolic differences of NSUN2−/− cells rescued with the empty vector (‘e.v.’), K190M, or the NSUN2 protein shown as a PCA plot (M) or as Log2 FC differences of the significant different (p < 0.01 NSUN2 versus e.v.) metabolites (N). The underlying data for this figure can be found in S4 and S7 Data and S1 File. CHX, cycloheximide; OP-puro, O-propargyl-puromycin; PCA, principle component analysis; RAPA, rapamycin; tRNA, transfer RNA. (TIF) [file pbio.3000297.s002.tif]

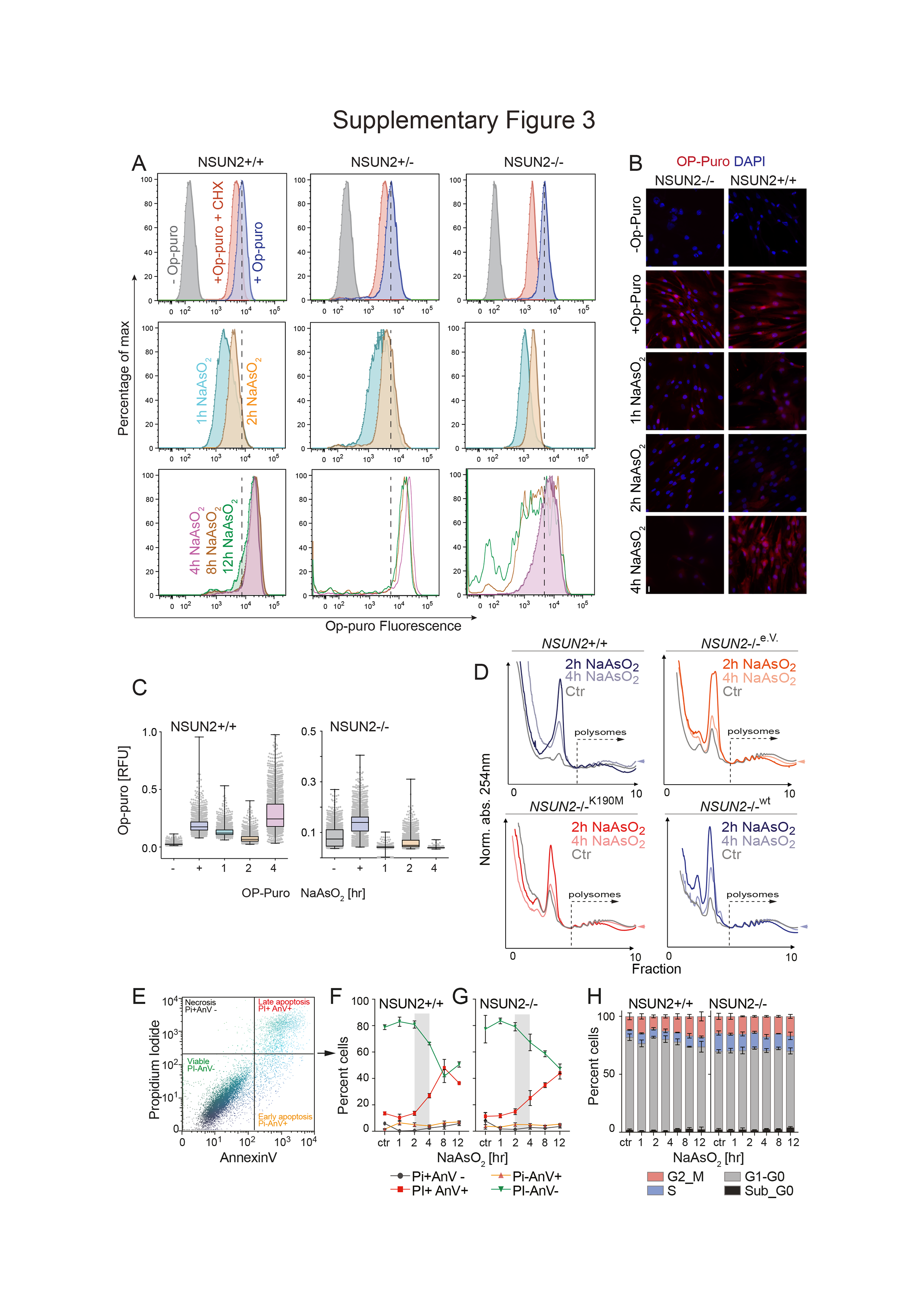

Supplement: S3 Fig — (A) Example raw data outputs from OP-puro fluorescence analysis using a flow cytometer for human dermal fibroblasts treated with sodium arsenite. Dotted line represents the mean level of OP-puro positive control. (B) Immunofluorescence detection of OP-puro incorporation in human dermal fibroblasts. DAPI: nuclear counterstain. Scale bar: 20 μm. (C) Measurement of OP-puro fluorescence intensity in cells using microscope-acquired images. Each dot represents one cell. Data are represented as median. (D) Second replicate of polysome profiling of NSUN2+/+ and NSUN2−/− cells rescued with wt or mutated NSUN2 (K190M). The empty vector (‘e.V.’)-infected cells served as control (see Fig 3F–3I). (E) Example of raw data output from AnV and PI analysis to measure cell death. (F, G) Percentage of cells that are viable, apoptotic, or necrotic in NSUN2+/+ and NSUN2−/− cells exposed to sodium arsenite for the indicated hours (hr) (n = 3 samples per time point). (H) Summary of cell cycle distribution shown in Fig 3A–3D. Data represented as mean in (K-H). Error bars are ±SD. The underlying data for this figure can be found in S1 File. AnV, AnnexinV; OP-puro, O-propargyl-puromycin; PI, propidium iodide; wt, wild-type. (TIF) [file pbio.3000297.s003.tif]

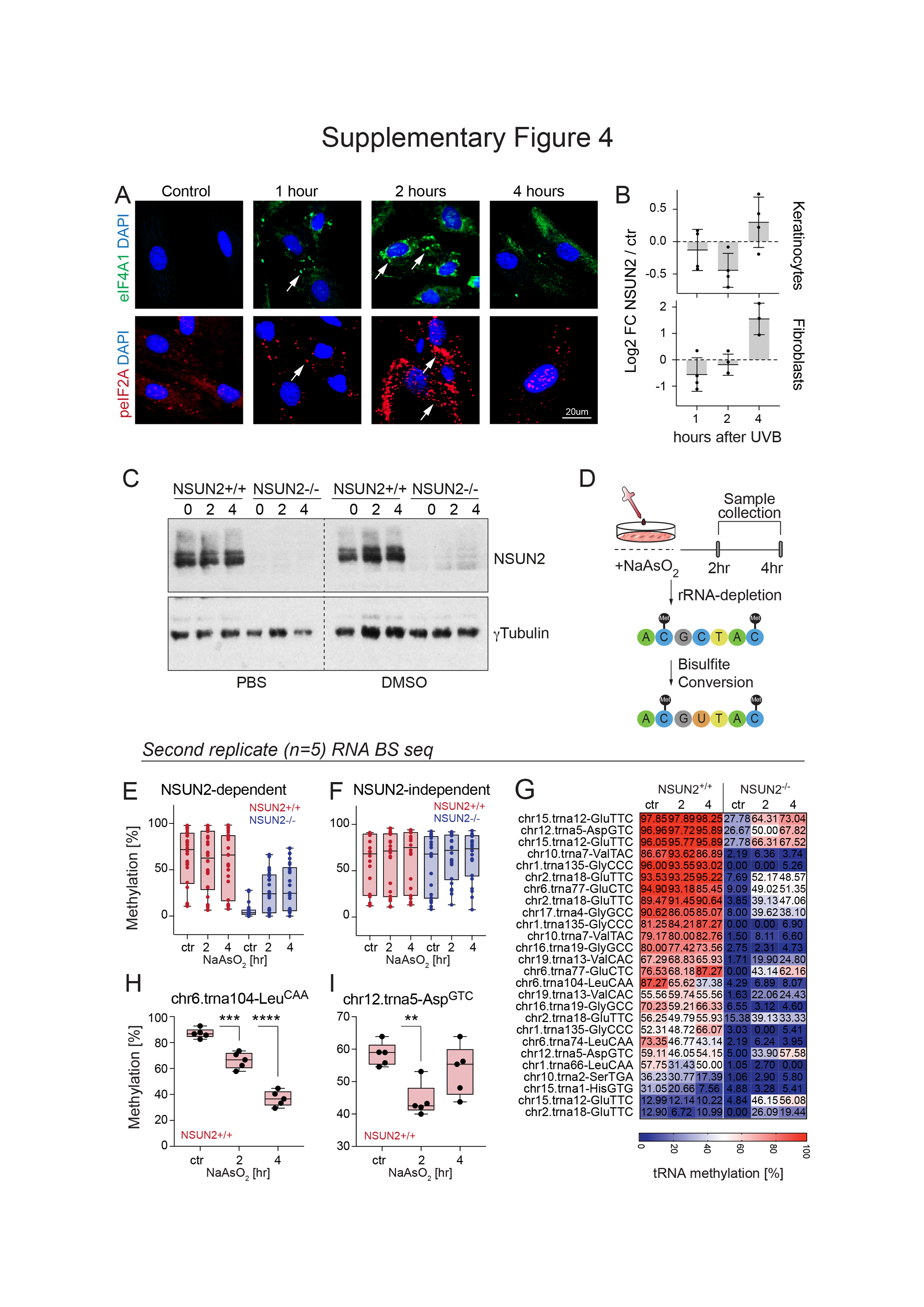

Supplement: S4 Fig — (A) Immunofluorescence detection of the stress granules markers eIF4A1 (upper panels) and p-eIF2A (lower panels) in untreated (control) or sodium arsenite–treated NSUN2+/+ and NSUN2−/− cells. DAPI: nuclear counterstain. Scale, 20 μm. (B) Nsun2 RNA levels in response to UVB exposure in primary human keratinocytes and dermal fibroblasts. (C) Western blot for NSUN2 in NSUN2+/+ and −/− cells incubated with vehicle control (DMSO, PBS). (D) Experimental outline of sample collection and RNA BS sequencing. (E,F) Quantification of tRNA methylation percentage of NSUN2-dependent (E) and -independent (F) sites in a second independent experiment (n = 5 samples per time point). (G) Second independent RNA BS-seq data shown as heatmap of methylation status of individual tRNA molecules in NSUN2+/+ and NSUN2−/− cells. (H, I) Quantification of methylation changes in the tRNAs LeuCAA and AspGTC in NSUN2+/+ and NSUN2−/− cells shown in (E). Data represented as median in (E, F, H, I). One-way ANOVA adjusted p-value (H,I), **p < 0.005, ***p < 0.0005, ****p < 0.0001. The underlying data for this figure can be found in S8 Data and S1 File. BS, bisulfite; BS-seq, BS sequencing; eIF2, eukaryotic Initiation Factor 2; tRNA, transfer RNA. (TIF) [file pbio.3000297.s004.tif]

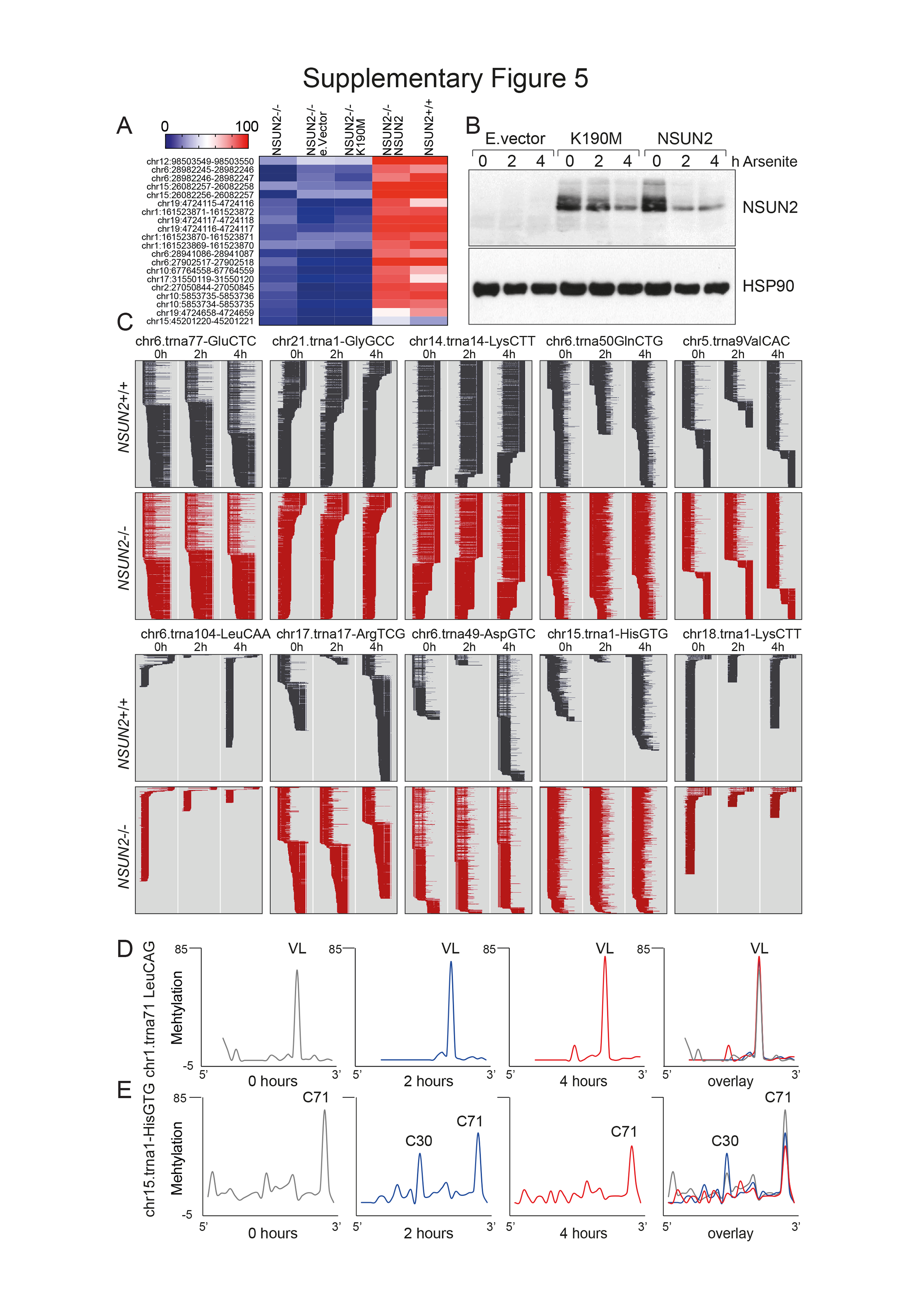

Supplement: S5 Fig — (A) Reexpression of NSUN2 but not K190M in NSUN2−/− cells restores methylation to similar levels of endogenous NSUN2 (NSUN2+/+). (B) Western blot for NSUN2 in NSUN2−/− cells infected with an empty (‘E.’) vector control, the enzymatic dead K190M, or the wild-type NSUN2 construct in cells untreated (0) or treated for 2 and 4 hours (h) with sodium arsenite. HSP90 served as a loading control. (C) Raw data (reads) for the indicated tRNAs obtained from small RNA sequencing in NSUN2-expressing (+/+; black) or -lacking (−/−; red) untreated (0h) or (h) treated 2 and 4 hours with arsenite. (D, E) Methylation levels (pooled from 5 replicates) of cytosines along tRNA 71-Leu CAG (C) and 1-His GTG (F) detecting m5C sites, in the variable loop (D) and C70 (E). The underlying data for this figure can be found in S10 Data and S1 File. HSP90, heat shock protein 90; m5C, 5-methylcytosine; tRF, tRNA-derived fragment; tRNA, transfer RNA. (TIF) [file pbio.3000297.s005.tif]

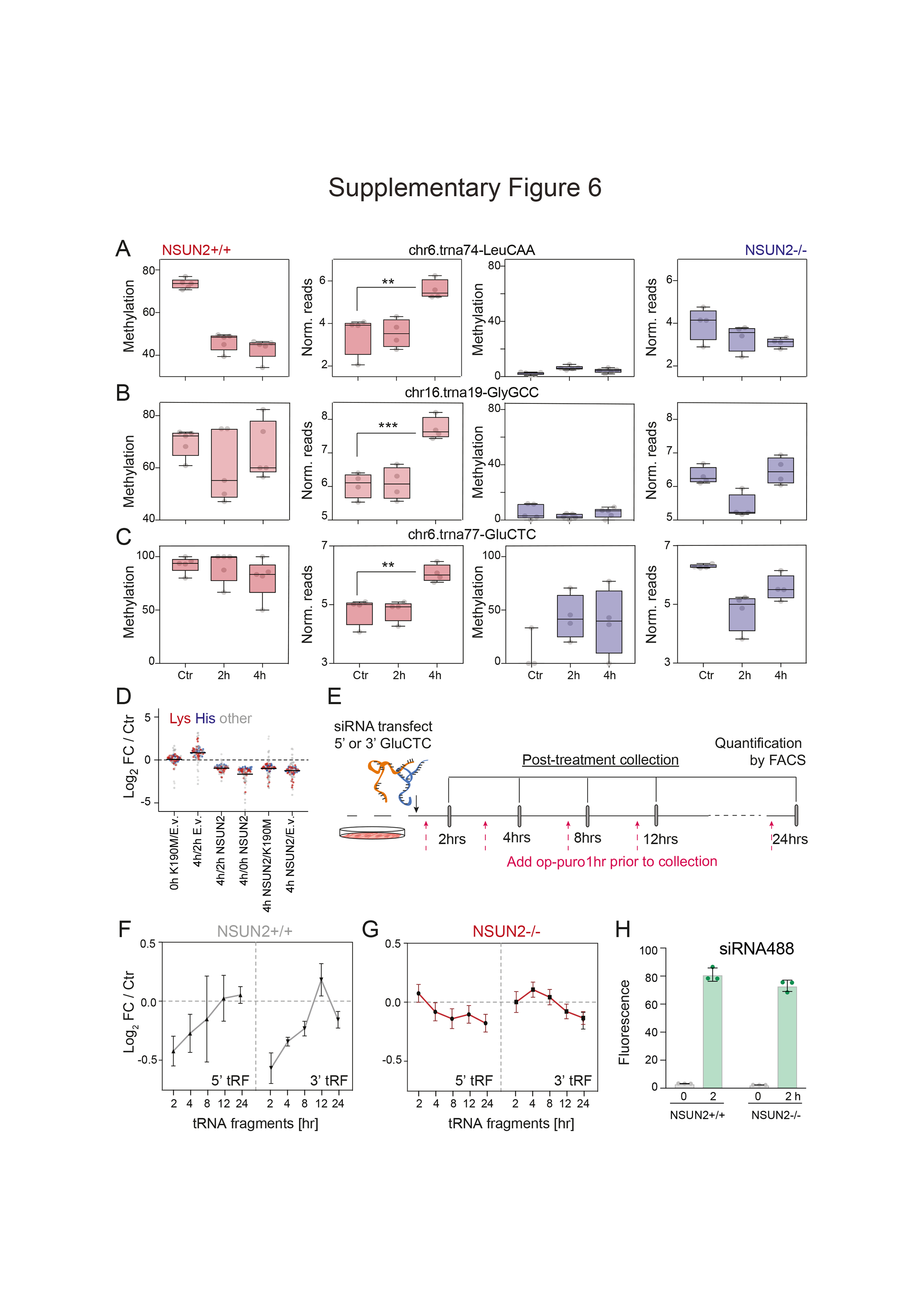

Supplement: S6 Fig — (A-C) Comparison of site-specific tRNA methylation and fragmentation in NSUN2+/+ (red) and NSUN2−/− (blue) cells. (n = 4 samples per time point). Data represent median and range. Adjusted p-value: one-way ANOVA. **p < 0.005, ***p = 0.0005. (D) Log2 FC of the down-regulated tRFs when NSUN2-overexpressing cells are exposed to stress for 4 hours. tRNA lysine-derived tRFs are highlighted in red; tRNA histidine-derived tRFs are highlighted in blue. Line indicates the mean. (E) Treatment regime to measure global protein synthesis of NSUN2+/+ and −/− cells transfected with tRNA GluCTC-derived 5′ and 3′ tRFs after exposure to sodium arsenite. (F, G) Log2 FC of protein synthesis in NSUN2+/+ (F) and NSUN2−/− (G) in response to synthetic 5′ or 3′ tRFs. (n = 3 samples per time point). (H) A fluorescence siRNA was used as a control for transfection efficiency. The underlying data for this figure can be found in S11 and S12 Data and the S1 File. FC, fold-change; siRNA, small interfering RNA; tRF, tRNA-derived fragment; tRNA, transfer RNA. (TIF) [file pbio.3000297.s006.tif]

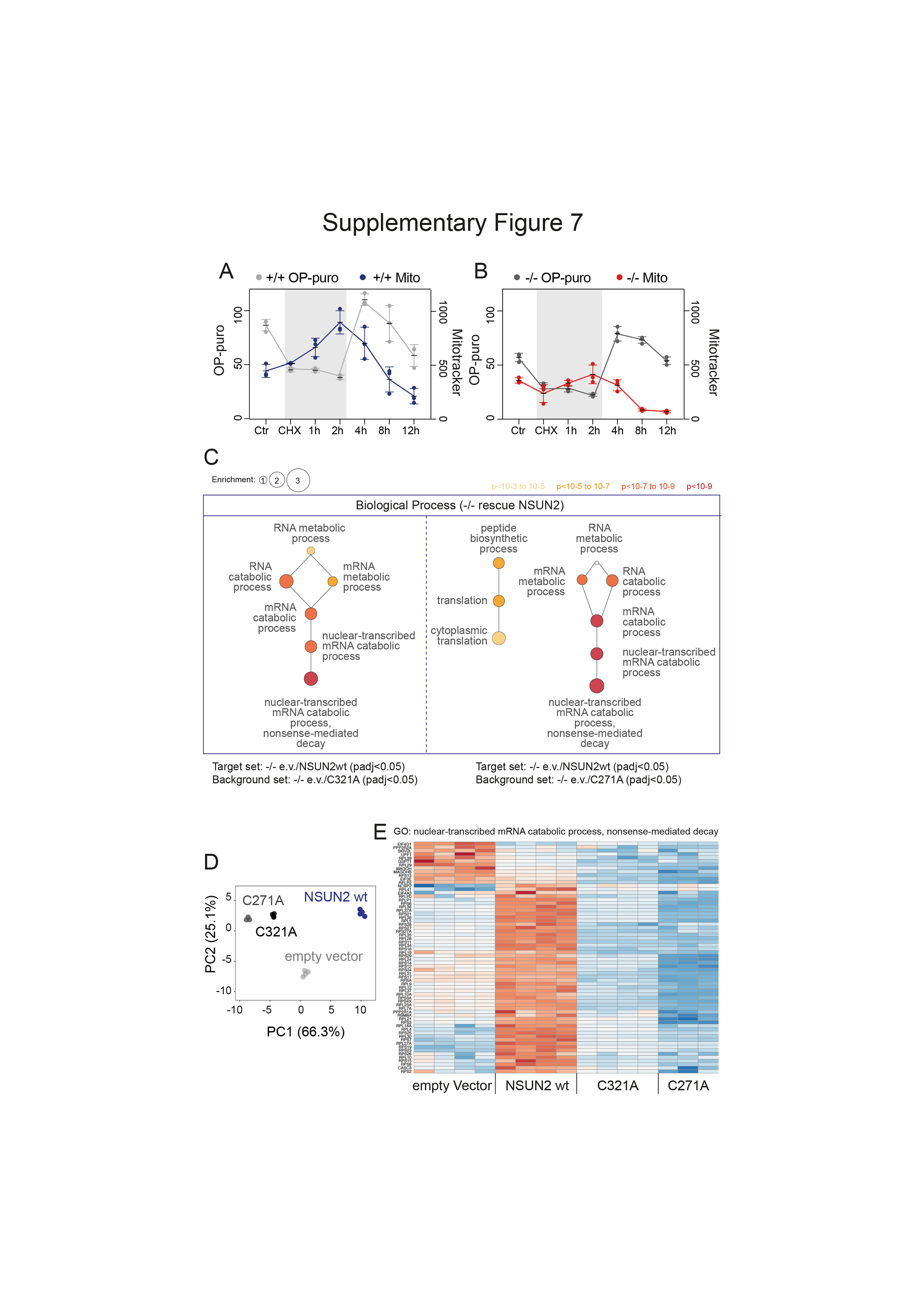

Supplement: S7 Fig — (A, B) Mitochondrial activity (‘mito’) and protein synthesis (‘OP-puro’) after exposure to arsenite for the indicated time (hours) or CHX in NSUN2+/+ (A) and NSUN2−/− (B) cells. (C) GO analyses using Ribo seq data in NSUN2−/− cells rescued with NSUN2 or the enzymatic dead versions of NSUN2 C321A (left panel) and C271A (right panel). (D, E) PCA plot (D) and heatmap (E) of genes belonging to the GO: nuclear-transcribed mRNA catabolic process, nonsense-mediated decay. The underlying data for this figure can be found in S15 Data and S1 File. CHX, cycloheximide; GO, Gene Ontology; OP-puro, O-propargyl-puromycin; PCA, principle component analysis; seq, sequencing. (TIF) [file pbio.3000297.s007.tif]
